# CircDLST promotes the tumorigenesis and metastasis of gastric cancer by sponging miR-502-5p and activating the NRAS/MEK1/ERK1/2 signaling

**DOI:** 10.1186/s12943-019-1015-1

**Published:** 2019-04-05

**Authors:** Jing Zhang, Lidan Hou, Rui Liang, Xiaoyu Chen, Rui Zhang, Wei Chen, Jinshui Zhu

**Affiliations:** 1 Department of Gastroenterology, Shanghai Jiao Tong University Affiliated Sixth People’s Hospital, No. 600 Yishan Road, Shanghai, 200233 China; 2 Department of Gastroenterology, Shanghai Ninth People’s Hospital, Shanghai Jiao Tong University School of Medicine, Shanghai, China

**Keywords:** circDLST, miR-502-5p, NRAS, Growth, Metastasis, Gastric cancer

## Abstract

**Background:**

Accumulating evidence shows that, the dysregulation of circular RNAs (circRNAs) is associated with the progression of multiple malignancies. But, the underlying mechanisms by which has_circ_0032627 (circDLST) contributed to gastric cancer (GC) remain undocumented.

**Methods:**

The expression and cellular localization of circDLST and its association with clinicopathological characteristics and prognosis in patients with GC was analysed by using fluorescence in situ hybridization. Gain- and loss-of-function experiments as well as a subcutaneous xenograft tumor model and a liver metastasis model from orthotopic implantation of GC tissues were conducted to assess the role of circDLST in GC cells. CircDLST specific binding with miR-502-5p was confirmed by dual luciferase gene report, RNA immunoprecipitation (RIP) assays and RIP-miRNA expression profiling. qRT-PCR and Western blot analysis was used to detect the effects of circDLST on miR-502-5p-mediated NRAS/MEK1/ERK1/2 signaling in GC cells.

**Results:**

The expression levels of circDLST were dramatically elevated in GC tissues as compared with the adjacent normal tissues, and acted as an independent prognostic factor of poor survival in patients with GC. Knockdown of circDLST inhibited the cell viability, colony formation, DNA synthesis, cell invasion and liver metastasis in vitro and in vivo, whereas overexpression of circDLST had the opposite effects. Furthermore, circDLST was co-localized with miR-502-5p in the cytoplasm of GC cells, and acted as a sponge of miR-502-3p in GC cells, which abrogated the tumor promoting effects of circDLST by inactivating the NRAS/MEK1/ERK1/2 signaling in GC cells.

**Conclusion:**

CircDLST promotes the tumorigenesis and metastasis of GC cells by sponging miR-502-5p to activate the NRAS/MEK1/ERK1/2 signaling.

**Electronic supplementary material:**

The online version of this article (10.1186/s12943-019-1015-1) contains supplementary material, which is available to authorized users.

## Introduction

The incidence and mortality of gastric cancer (GC) rank the fifth place in tumors of digestive system worldwide [1] and it is the third leading cause of cancer-related deaths in China [2]. In spite of the decreased incidence of GC, most of the cases still harbor a poor prognosis when diagnosed duo to their tumor invasiveness and distant metastasis [3]. GC is a long-term progressive disease associated with the activation of pro-oncogenes or inactivation of tumor suppressors [4]. Thus, identification of novel candidate biomarkers may offer insights for the early detection of GC.

Considerable evidence validates that, the dysregulation of noncoding RNAs (ncRNAs) is associated with the initiation and pathogenesis of GC [5–7]. Circular RNAs (circRNAs), a new class of ncRNAs, have a covalently closed loop, display a tissue specific expression and are highly conserved owing to their resistance to RNase R [8]. They interact with RNA binding proteins involved in RNA translation [9], and facilitate the transcription of their parental genes [10, 11], of which circAGO2 promotes the tumor growth by regulating HuR-repressed AGO2 expression [12]. Moreover, circRNAs act as sponges of miRNAs [13], of which cdr1as leads to miRNA deregulation in brain diseases [14, 15], and circ-ITCH/circFBLIM1/circ_100290 act as sponges of miR-17/− 224/− 346/− 29 to rgulate tumor growth [16–18].

CircRNAs act in the progression and prognosis of GC. They are presented as the prognostic markers of GC. circ_0000520, circLARP4 and circPVT1 have been identified as potential biomarkers for predicting the survival of patients with GC [19–21]. They also act as oncogenes or tumor suppressors in GC cells. circLARP4 and circFAT1(e2) impede the proliferation and invasion of GC cells [20, 22], while circPVT1 and circRNA7690–15 display a carcinogenic effect [21, 23]. CircRNAs function as miRNA sponges in GC cells, of which circLARP4/circ_100269 act as sponges of miR-424/− 630 to restrain GC growth [20, 24].

We previously proposed that, circLARP4 is a tumor suppressor in GC by sponging miR-424 [20]. Herein, we identified a novel oncogenic circ_0032627 derived from the linear gene DLST (termed as circDLST), and found that, circDLST promoted the tumorigenesis and metastasis of GC cells by sponging miR-502-5p and activating the NRAS/MEK1/ERK1/2 signaling; High expression of circDLST acted as an independent prognostic factor of poor survival in patients with GC.

## Materials & methods

### Clinical data

The clinicopathological data for 396 cases of GC patients and 42 adjacent normal tissues as well as the relative expression levels of miRNAs (has-miR-502-5p, has-miR-193b-5p, has-miR-542-3p, has-miR-362-5p and has-miR-203a-5p) were downloaded from The Cancer Genome Atlas (TCGA) RNA-seq database (https://genome-cancer.ucsc.edu). A tissue microarray (TMA) containing 71 paired GC tissues (Cat No. STC1601) was purchased from the shanghai Superbiotek Pharmaceutical Technology Co., Ltd. (Shanghai, China). The protocols used in this study were approved by the Ethics Committee of Shanghai Sixth People’s Hospital. The specimens were classified according to the TNM staging, and diagnosed by two independent pathologists.

### Identification of circDLST specific binding with miRNAs

The circDLST specific binding with miRNAs (has-miR-502-5p, has-miR-193b-5p, has-miR-542-3p, has-miR-362-5p and has-miR-203a-5p) was identified by using the circRNA expression profiling and miRbase database (http://www.mirbase.org /index.shtml) in GC cells. The target genes of miR-502-5p were screened by using mirPath v.3 (http://snf-515788.vm.okeanos.grnet.gr/) and microT_CDS (http://diana.imis.athena-innovation.gr/DianaTools/index.php?r=microT_CDS/index).

### Cell culture

Gastric epithelial cell line GES-1 and GC cell lines (MGC-803, BGC-823, SGC-7901, HGC-27, AGS, MKN-45 and MKN-28) were stored in Digestive Disease Laboratory of our hospital. They were cultured in Dulbecco’s Modified Eagle medium (DMEM) medium supplemented with 10% heat-inactivated fetal bovine serum (FBS), 100 U/ml of penicillin, and 100 μg/ml of streptomycin (HyClone) in a humidified atmosphere containing 5% CO_2_ at 37 °C. All cell lines were used for the functional experiments in 6 months.

### Fluorescence in situ hybridization (FISH)

Digoxin-labeled probe sequences for hsa_circ_0032627 (5′-ACAGCTGTAGTTCTG AAAAAGCGAACACTGAAGACACTGTTGTTAATGCTTTCTCCCACCTGACATCTCCCTCTGTGACAGATTCTGCAAACGCTGGGG-3′) were used for analysis of the expression levels and localization of circDLST in GC tissues. Digoxin-labeled probe sequences for hsa_circ_0032627 (5′-CACTGTTGTTAATGCTTTCTCCCACC TG-3‘) and hsa-miR-502-5p (5’-TAGCACCCAGATAGCAAGGAT-3′) were used for analysis of the co-localization of circDLST and miR-502-5p in GC cells (MKN-45 and MKN-28). The detailed description of FISH analysis was performed as previously reported [20]. The analysis software Image-pro plus 6.0 (Media Cybernetics, Inc., Rockville, MD, USA) was used to analyse the immunofluorescence accumulation optical density (IOD) of circDLST and miR-502-5p in GC tissues.

### Quantitative real-time PCR (qRT-PCR)

Total RNA was extracted by using TRIzol, reverse transcription was performed by using M-MLV and cDNA amplification by using the SYBR Green Master Mix kit (Takara, Otsu, Japan). Total RNA was isolated using a High Pure miRNA isolation kit (Roche) and RT-PCR using a TaqMan MicroRNA Reverse Transcription kit (Life Technologies). The primers were listed in Additional file 1: Table S1.

### Western blot analysis

GC cell lines (MKN-28 and BGC-823) were harvested and extracted by using lysis buffer. Cell extracts were boiled in loading buffer and equal amount of cell extracts were separated on 15% SDS-PAGE gels. Separated protein bands were transferred into polyvinylidene fluoride membranes. The primary antibodies anti-NRAS (CY5549, Rabbit IgG antibody, Abways, Shanghai, China), anti-MEK1 (CY5042, Rabbit IgG antibody, Abways, Shanghai, China), anti-Phospho-MEK1 (S298) Antibody (CY5854, Rabbit IgG antibody, Abways, Shanghai, China), anti-ERK1/2 (CY5487, Rabbit IgG antibody, Abways, Shanghai, China), anti-Phospho-ERK1/2 (CY5277, Rabbit IgG antibody, Abways, Shanghai, China), anti-PCNA (AB0051, Rabbit IgG antibody, Abways, Shanghai, China), anti-MMP2 (CY5189, Rabbit IgG antibody, Abways, Shanghai, China) and anti-GAPDH (#5174, Rabbit antibody, CST, Shanghai, China) were diluted at a ratio of 1:1000 according to the instructions and incubated overnight at 4 °C.The detailed description of Western blot analysis was performed as previously reported [20].

### Luciferase reporter assay

MKN-45, MKN-28 and BGC-823 cell lines were seeded into 96-well plates and were co-transfected with PRL-TK-pMIR-circDLST or PRL-TK-pMIR-NRAS 3’UTR, and miR-502-5p mimic or miR-NC. After 48 h of incubation, the firefly and Renilla luciferase activities were detected with a dual-luciferase reporter assay (Promega, Madison, WI, USA).

### Plasmid, shRNA, miRNA mimic and inhibitor

Plasmid mediated circDLST vector, shRNA targeting circDLST vector (sh-circDLST, 5′-CAGGUGGGAGAAAGCAUUATT-3′), siRNA targeting NRAS gene (si-NRAS, 5′-GCGCACTGACAATCCAGCTAATCCA-3′), miR-502-5p mimic, inhibitor and NRAS plasmid were purchased from GenePharma (Shanghai, China). The negative control (NC), sh-NC, pEX-3 (Vector) or miR-NC was used as the control vectors. GC cell lines were planted in 6-well plates 24 h prior to sh-circDLST, circDLST, miR-502-5p mimic or inhibitor transfection with 50–60% confluence, and then were transfected with Lipofectamine 2000 (Invitrogen, Carlsbad, CA, USA) according to the manufacture instructions.

### MTT, colony formation, EdU and Transwell assays

MTT, colony formation, 5-ethynyl-2-deoxyuridine (EdU) and Transwell assays were performed as previously reported [20].

### Wound-healing assay

Cell migration was assessed by using the wound-healing assay, and the detailed description was performed as previously reported [5].

### RNase R treatment

Total RNA (2 μg) was incubated for 30 min at 37 °Cwith 3 U/μg of RNase R (Epicentre Technologies, Madison, WI, USA). After GC cell lines were treated with RNase R, the expression levels of DLST and circDLST were detected by qRT-PCR analysis.

### RNA immunoprecipitation (RIP)

RIP assay was performed in MKN-45 and MKN-28 cell lines by using a Magna RIP RNA-binding protein Immunoprecipitation Kit (Millipore) according to the manufacturer’s instructions. Antibodies for RIP assays against Ago2 and IgG were purchased from Abcam (ab5072, Rabbit polyclonal antibody, Cambridge, MA, USA).

### RIP-miRNA expression profiling

The miRNAs were pulled-down from Ago2 and IgG-expressed MKN-45 cells and hybridized on miRNA microarray chip containing human miRNA probes found in the miRNA Registry. Microarray chip analysis was conducted by KANGCHEM (Shanghai, PR, China).

### In vivo tumorigenesis assay

Male nude mice (6 weeks old) were purchased from Shanghai SIPPR-BK Laboratory Animal Co. Ltd. (Shanghai, China) and maintained in microisolator cages. All the animals were conducted according to the institutional guidelines, and approved by the Animal Ethics Committee of our hospital. The mice were subcutaneously inoculated with 5 × 10^7^ of BGC-823 cells stably transfected with circDLST/vector or MKN-45 cells stably transfected with sh-circDLST/sh-NC that had been resuspended in PBS with 50% Matrigel. The body weight and tumor size were measured every other day, and the tumor volume was calculated depending on the formula: length × width^2^/2.

### Live metastasis model

A sh-circDLST/sh-NC stably transfected MKN-45 cell line was subcutaneously injected and maintained by passage in the hypodermis of nude mice. Live metastasis model was performed by using the orthotopic implantation of histological intact MKN-45 tumor tissues. The detailed description of live metastasis model was performed as previously reported [25].

### Statistical analysis

Statistical analysis was conducted as previously reported [20].

## Results

### High expression of circDLST was associated with poor survival in patients with GC

**Fig. 1:**
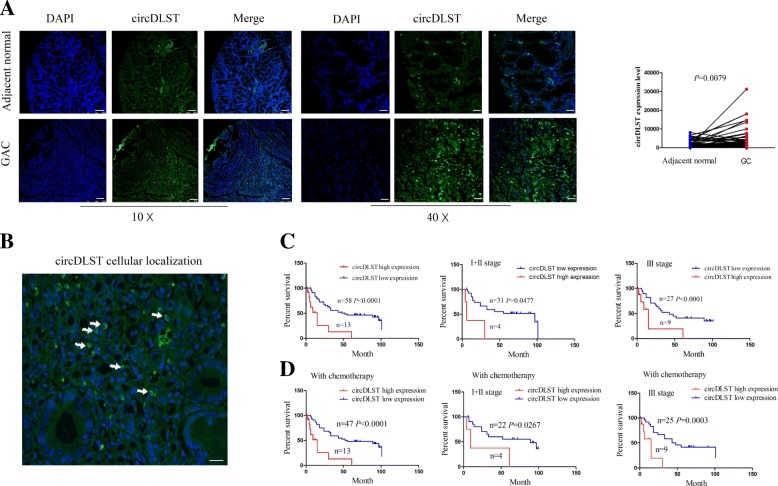
High expression of circDLST was associated with poor survival in patients with GC. **a** FISH analysis of the expression levels of circDLST in 71 paired GC tissue samples. **b** FISH analysis of the cellular localization of circDLST in GC tissue cells. **c** Kaplan Meier analysis of the association of high or low circDLST expression with overall survival in patients with GC either in early or late stage. **d** Kaplan Meier analysis of the association of high or low circDLST expression with overall survival in early or advanced patients receiving chemotherapy. Bar scale: 125 μm

We previously confirmed an antitumor effect of toosendanin in GC cells [26], and identified a novel has_circ_0032627 derived from the linear gene DLST (termed as circDLST) as a key target of toosendanin in GC cells (data not shown). The expression levels of circDLST in GC tissues were detected by using FISH analysis, indicated that, circDLST expression levels were strikingly increased in GC tissues as compared with the adjacent normal tissues (*n* = 71, *P* = 0.0079; Fig. 1a), and predominantly localized in the cytoplasm of GC tissue cells (Fig. 1b).

According to circDLST expression levels, survival time and survival status, we obtained a cutoff value of circDLST, which divided the patients into circDLST high expression and circDLST low expression groups (Additional file 2: Figure S1). We then found that, high expression of circDLST had no association with the clinicopathological characteristics in patients with GC (each *P* > 0.05, Additional file 3: Figure S2 and Additional file 1: Table S2). Kaplan Meier analysis demonstrated that, the patients (*P* < 0.0001) either in stage I + II (*P* = 0.0477) or stage III (*P* < 0.0001) with circDLST high expression had a poorer survival as compared with those with circDLST low expression (Fig. 1c). We also analysed the association of chemotherapy (oxaliplatin+ 5-Fu) with the overall survival in patients with GC, and found that, the patients with chemotherapy had no difference in overall survival as compared with those without chemotherapy (*P* > 0.05, Additional file 4: Figure S3). But, the patients receiving chemotherapy (*P* < 0.0001) either in stage I + II (*P* = 0.0267) or stage III (*P* = 0.0003) with circDLST high expression possessed a poorer survival as compared with those with circDLST low expression (Fig. 1d).

A Cox proportional hazard model was used to analyse the association of circDLST with the prognosis of patients with GC. Univariate and multivariate analysis revealed that, circDLST high expression (*P* < 0.0001) as well as gender (*P* = 0.023) was an independent prognostic factor of poor survival in patients with GC (Additional file 1: Table S3).

### Knockdown of circDLST inhibited the proliferation and colony formation of GC cells

**Fig. 2:**
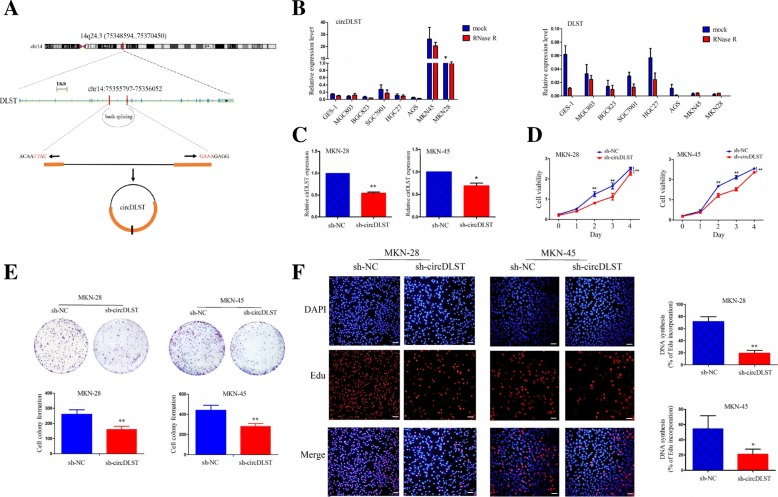
Knockdown of circDLST inhibited the proliferation and colony formation of GC cells. **a** The genomic loci of the DLST gene and circDLST. **b** qRT-PCR analysis of the expression levels of DLST and circDLST after treatment with RNase R in GC cell lines. **c** qRT-PCR analysis of the knockdown efficiency of sh-circDLST after the transfection for 48 h in MKN-28 and MKN-45 cells. **d** MTT analysis of the cell viability after the transfection of sh-circDLST or sh-NC in MKN-28 and MKN-45 cells. **e** Colony formation analysis of the cell colony number after the transfection of sh-circDLST or sh-NC in MKN-28 and MKN-45 cells. **f** EdU analysis of the DNA synthesis after the transfection of sh-circDLST or sh-NC in MKN-28 and MKN-45 cells. Bar scale: 125 μm. Data are the means ± SEM of three experiments. *P < 0.05; **P < 0.01

Has_circ_0032627 (chr14:75355797–75,356,052), derived from exon 4, 5 regions within dihydrolipoamide S-succinyltransferase (DLST) locus, is located on chromosome 14q24.3, and termed as circDLST, whose genomic sequence is 255 nt and spliced length is 128 nt. (Fig. 2a). qRT-PCR analysis showed that, after exposure to the RNase R, DLST expression levels were decreased, while circDLST displayed a resistance to RNase R exonuclease, indicating that, circDLST possessed a closed loop structure (Fig. 2b).

After sh-circDLST vector was transfected into MKN-28 and MKN-45 cell lines for 48 h, the knockdown efficiency of sh-circDLST was determined by qRT-PCR analysis (Fig. 2c). MTT and colony assays demonstrated that, knockdown of circDLST repressed the cell viability (*P* < 0.01, Fig. 2d) and colony formation abilities of MKN-28 and MKN-45 cells (*P* < 0.01, Fig. 2e). EdU incorporation assay indicated that, the DNA synthesis of MKN-28 and MKN-45 cells was retarded by knockdown of circDLST, as compared with the si-NC group (Fig. 2f).

### Knockdown of circDLST inhibited the migration and invasion of GC cells

**Fig. 3:**
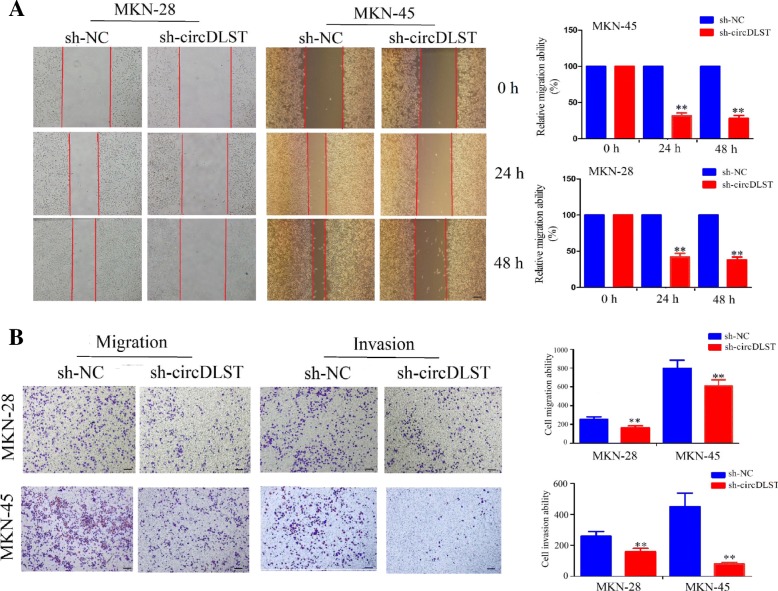
Knockdown of circDLST inhibited the migration and invasion of GC cells. **a** Wound-healing analysis of the cell migration after the transfection of sh-circDLST or sh-NC in MKN-28 and MKN-45 cells. **b** Transwell analysis of the cell migration and invasive potential after the transfection of sh-circDLST or sh-NC in MKN-28 and MKN-45 cells. Bar scale: 125 μm. Data are the means ± SEM of three experiments. **P < 0.01

After the sh-circDLST vector was transfected into MKN-28 and MKN-45 cells, the wound-healing assay showed that, knockdown of circDLST suppressed the cell migration of MKN-28 and MKN-45 cells in a time dependent manner (*P* < 0.01, Fig. 3a). Transwell migration and invasion assays indicated that, the cell migration and invasion capabilities of MKN-28 and MKN-45 cells were markedly weakened by knockdown of circDLST, as compared with the si-NC group (*P* < 0.01, Fig. 3b).

### Overexpression of circDLST promoted the proliferation and invasion of GC cells

**Fig. 4:**
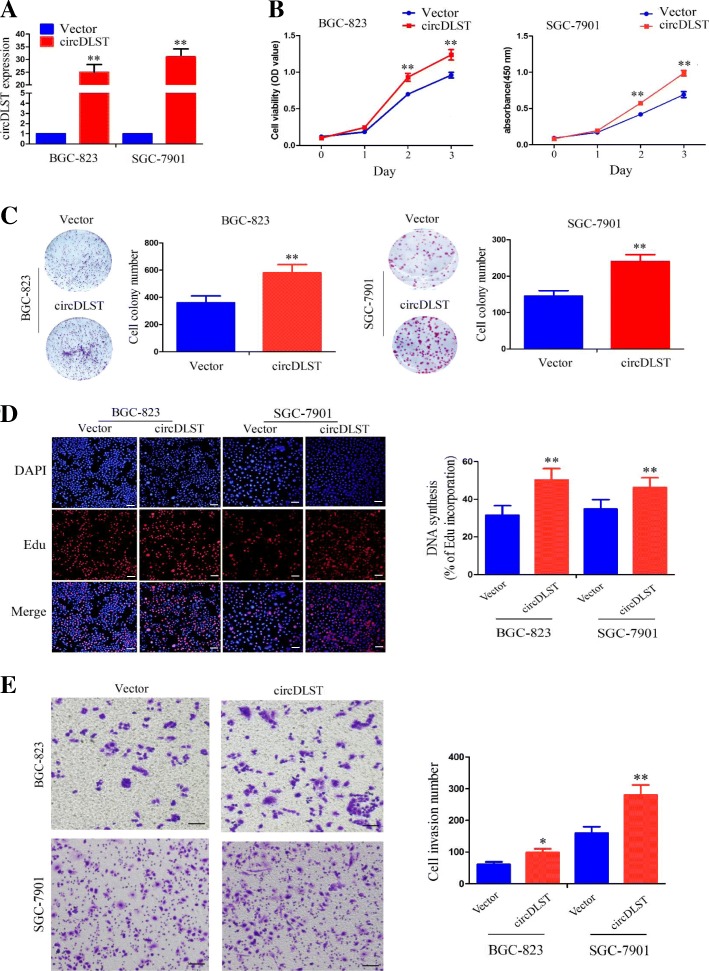
Overexpression of circDLST promoted the proliferation and invasion of GC cells. **a** qRT-PCR analysis of the transfection efficiency of circDLST plasmid in BGC-823 and SGC-7901 cells. **b** MTT analysis of the cell viability after the transfection of circDLST or vector in BGC-823 and SGC-7901 cells. **c** Colony formation analysis of the cell colony number after the transfection of circDLST or vector in BGC-823 and SGC-7901 cells. **d** EdU analysis of the DNA synthesis after the transfection of circDLST or vector in BGC-823 and SGC-7901 cells. **e** Transwell analysis of cell invasive potential after the transfection of circDLST or vector in BGC-823 and SGC-7901 cells. Bar scale: 125 μm. Data are the means ± SEM of three experiments. *P < 0.05; **P < 0.01

After circDLST plasmid was transfected into BGC-823 and SGC-7901 cell lines for 48 h, the transfection efficiency of circDLST was ascertained by qRT-PCR analysis (*P* < 0.01, Fig. 4a). MTT and colony formation assays showed that, overexpression of circDLST promoted the cell viability (*P* < 0.01, Fig. 4b) and colony formation abilities of BGC-823 and SGC-7901 cells (*P* < 0.01, Fig. 4c). EdU and Transwell assays revealed that, both of the DNA synthesis (*P* < 0.01, Fig. 4d) and invasion capabilities (Fig. 4e) of BGC-823 and SGC-7901 cells were enhanced by overexpression of circDLST, as compared with the control group.

### The effects of circDLST on in vivo tumor growth and liver metastasis

**Fig. 5:**
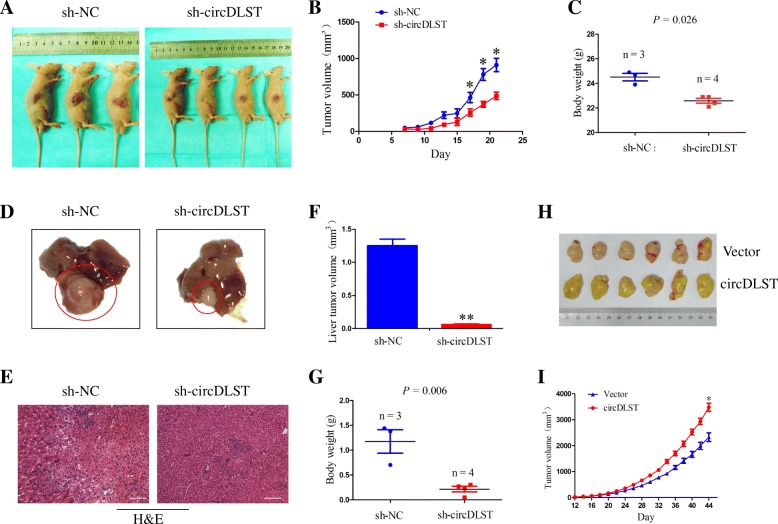
The effects of circDLST on in vivo tumor growth and liver metastasis. **a** Comparison of the tumor size of xenograft tumors induced by sh-circDLST and sh-NC transfected MKN-45 cells. **b** A growth curve analysis of the tumor growth in sh-circDLST and sh-NC transfected MKN-45 groups. **c** Comparison of the body weight in sh-circDLST and sh-NC transfected MKN-45 groups. **d** Comparison of the liver metastatic tumors from sh-circDLST and sh-NC transfected gastric tumor tissues. **e** HE staining of the metastatic tumor cells in liver tissues in sh-circDLST and sh-NC groups. **f, g** Comparison of the liver tumor size and body weight in sh-circDLST and sh-NC groups. **h** Comparison of the tumor size of xenograft tumors induced by circDLST or vector transfected BGC-823 cells. **i** A growth curve analysis of the tumor growth in circDLST or vector transfected BGC-823 groups. Bar scale: 250 μm. Data are the means ± SEM of three experiments. *P < 0.05

To elucidate whether circDLST affected in vivo tumor growth and liver metastasis, we constructed a sh-circDLST or sh-NC stably transfected MKN-45 cell line, which was then subcutaneously injected into the flank of nude mice. After an observation for 21 days, we found that, the volumes of xenograft tumors induced by sh-circDLST transfected MKN-45 cells were smaller than those induced by NC transfected cells (Fig. 5a). A growth curve indicated that, the tumors in sh-circDLST group turned slow in a time dependent manner (*P* < 0.05, Fig. 5b), and the body weight was lightened in sh-circDLST transfected group as compared with the sh-NC group (*P* = 0.026, Fig. 5c). The orthotopic implantation of GC tissues formed by sh-circDLST or sh-NC transfected MKN-45 cells were used to establish a liver metastasis model, indicating that, knockdown of circDLST inhibited the liver metastasis of gastric tumors as compared with the sh-NC group (Fig. 5d), and HE staining confirmed a decreased metastatic tumor cells in live tissues by knockdown of circDLST (Fig. 5e). The live tumor volume (*P* < 0.01, Fig. 5f) and body weight (*P* = 0.006, Fig. 5g) were lowered in sh-circDLST transfected group as compared with the sh-NC group. We also set up a circDLST or vector stably transfected BGC-823 cell line, and found that, the volumes of xenograft tumors induced by circDLST transfected BGC-823 cells were larger than those by NC transfected cells (Fig. 5h), and the tumors in circDLST transfected group showed a rapid growth as compared with the vector group (*P* < 0.05, Fig. 5i).

### CircDLST acted as a sponge of miR-502-5p in GC cells

**Fig. 6:**
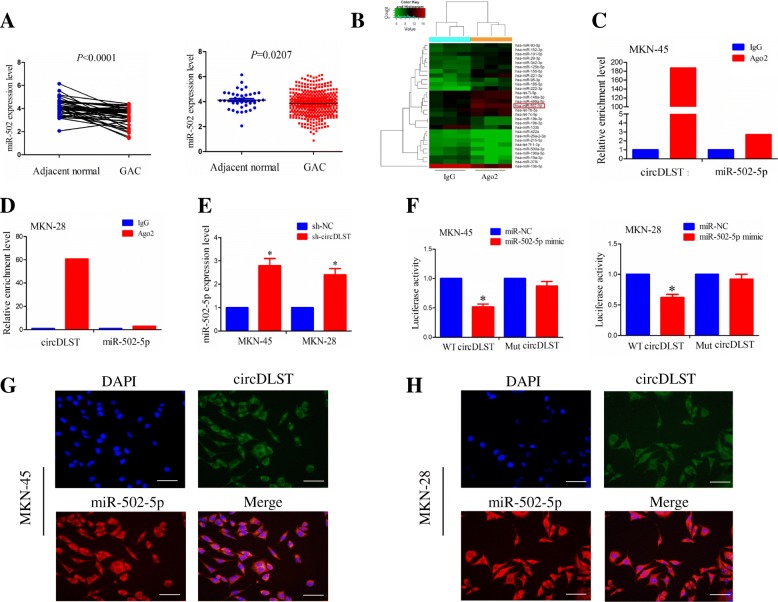
CircDLST acted as a sponge of miR-502-5p in GC cells. **a** TCGA analysis of the expression levels of miR-502-5p in paired and unpaired GC tissues. **b** miRNA expression profiling of miR-502-5p pulled down from the Ago2 or IgG-expressed MKN-45 cells. **c, d** RIP and qRT-PCR analysis of the amount of circDLST and miR-502-5p pulled down from Ago2 or IgG expressed MKN-45 and MKN-28 cells. **e** qRT-PCR analysis of the effects of circDLST on miR-502-5p expression levels in MKN-45 and MKN-28 cells. **f** The luciferase activity of WT and Mut circDLST after co-transfection with PRL-TK-pMIR-Luc-circDLST and miR-502-5p mimic or miR-NC in MKN-45 and MKN-28 cells. **g, h** FISH analysis of the co-localization of circDLST and miR-502-5p in MKN-45 and MKN-28 cells. Bar scale: 125 μm. Data are the means ± SEM of three experiments. *P < 0.05

According to the circRNA expression profiling and miRbase, circDLST specific binding with miRNAs (has-miR-502-5p, has-miR-193b-5p, has-miR-542-3p, has-miR-362-5p and has-miR-203a-5p) was identified in GC cells (Additional file 5: Figure S4). TCGA cohort showed that, only miR-502-5p expression levels were decreased in paired (*P* < 0.0001) and unpaired GC tissues (*P* = 0.0207, Fig. 6a) rather than other miRNAs (Additional file 6: Figure S5). Then, we gained a cutoff of miR-502-5p and divided the patients into high miR-502-5p expression and low miR-502-5p expression groups (Additional file 7: Figure S6A), but found that, the patients with low miR-502-5p expression had no difference in overall survival (*P* = 0.0846) and tumor recurrence (*P* = 0.4190) as compared with those with high miR-502-5p expression (Additional file 7: Figure S6B).

A online circular RNA interactome database revealed the Ago2 occupancy in the region of circDLST (Additional file 1: Table S4). We identified an increased enrichment of miR-502-5p, pulled down from Ago2-expressed MKN-45 cells as compared with those pulled-down from IgG-expressed MKN-45 cells by using a RIP-miRNA expression profiling (Fig. 6b, Additional file 1: Table S5). RIP assay was performed for Ago2 in MKN-45 and MKN-28 cells and assessed the expression levels of endogenous circDLST and miR-502-5p pulled-down from Ago2-expressed MKN-45 and MKN-28 cells by qRT-PCR analysis, indicating that, circDLST and miR-502-5p were highly enriched in the Ago2 pellet as compared with those in the input control (Fig. 6c, d).

We estimated the effects of circDLST on miR-502-5p expression levels by qRT-PCR analysis, which indicated that, knockdown of circDLST increased the expression levels of miR-502-5p in MKN-45 and MKN-28 as compared with the sh-NC group (*P* < 0.05, Fig. 6e). PRL-TK-pMIR-Luc reporter containing the wild type (WT) or mutant (MUT) circDLST (Additional file 5: Figure S4) was co-transfected with miR-502-5p mimic into MKN-45 and MKN-28 cells. The results showed that, miR-502-5p mimic decreased the luciferase activity of WT circDLST in these two cell lines (*P* < 0.05), but had no effects on that of Mut circDLST, as compared with miR-NC group (Fig. 6f). FISH analysis demonstrated that, circDLST and miR-502-5p was co-localized in the cytoplasm of MKN-45 and MKN-28 cells (Fig. 6g, h). qRT-PCR analysis was conducted to examine the expression levels of miR-502-5p in GC cell lines and Pearson correlation analysis revealed that, circDSLT had a negative correlation with miR-502-5p expression in GC cell lines (r = − 0.782, *P* = 0.038; Additional file 8: Figure S7).

### MiR-502-5p reversed the tumor promoting effects of circDLST in GC cells by regulating the NRAS/MEK1/ERK1/2 signaling

**Fig. 7:**
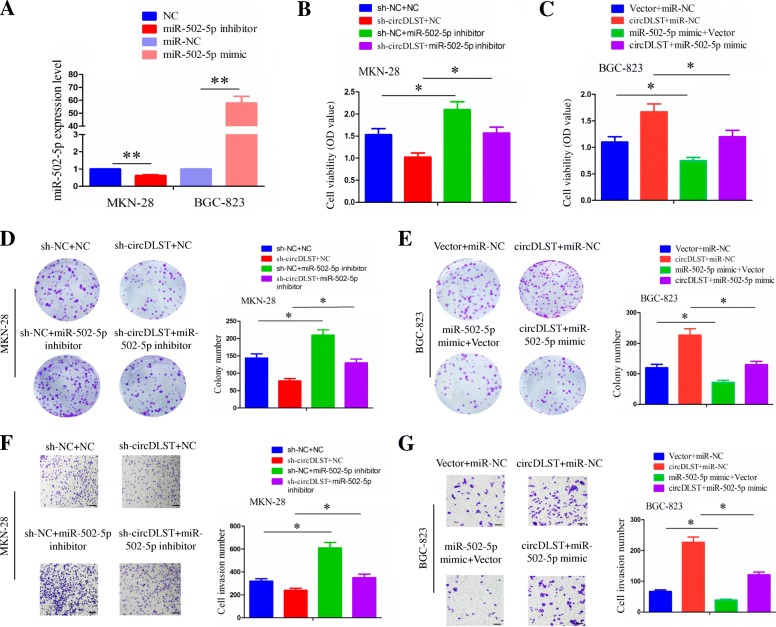
MiR-502-5p reversed the tumor promoting effects of circDLST in GC cells. **a** qRT-PCR analysis of the transfection efficiency of miR-502-5p inhibitor in MKN-28 cells or miR-502-5p mimic in BGC-823 cells. **b, c** MTT analysis of the cell viability after the co-transfection of miR-502-5p inhibitor and sh-circDLST in MKN-28 cells or miR-502-5p mimic and circDLST in BGC-823 cells. **d, e** Colony formation analysis of the colony number after the co-transfection of miR-502-5p inhibitor and sh-circDLST in MKN-28 cells or miR-502-5p mimic and circDLST in BGC-823 cells. **f, g** Transwell analysis of the cell invasive potential after the co-transfection of miR-502-5p inhibitor and sh-circDLST in MKN-28 cells or miR-502-5p mimic and circDLST in BGC-823 cells. Bar scale: 125 μm. Data are the means ± SEM of three experiments. *P < 0.05; **P < 0.01

To investigate the functional interaction between circDLST and miR-502-5p in MKN-28 and BGC-823 cells, we conducted the rescue experiments such as MTT, colony formation and Transwell assays. The transfection efficiency of miR-502-5p inhibitor or mimic was determined in MKN-28 or BGC-823 cells (*P* < 0.01, Fig. 7a). After the co-transfection of miR-502-5p inhibitor and sh-circDLST in MKN-28 cells or miR-502-5p mimic and circDLST in BGC-823 cells, we found that, miR-502-5p inhibitor promoted the cell proliferation (*P* < 0.05, Fig. 7b), colony formation (*P* < 0.05, Fig. 7d) and cell invasive potential (*P* < 0.05, Fig. 7f) in MKN-28 cells, while miR-502-5p mimic had the tumor suppressive effects in BGC-823 cells (*P* < 0.05, Fig. 7c, e, g). Moreover, miR-502-5p inhibitor reversed the tumor suppressive effects induced by knockdown of circDLST in MKN-28 cells (*P* < 0.05, Fig. 7b, d, f), while miR-502-5p mimic attenuated the tumor promoting effects induced by circDLST in BGC-823 cells (*P* < 0.05, Fig. 7c, e, g).

**Fig. 8:**
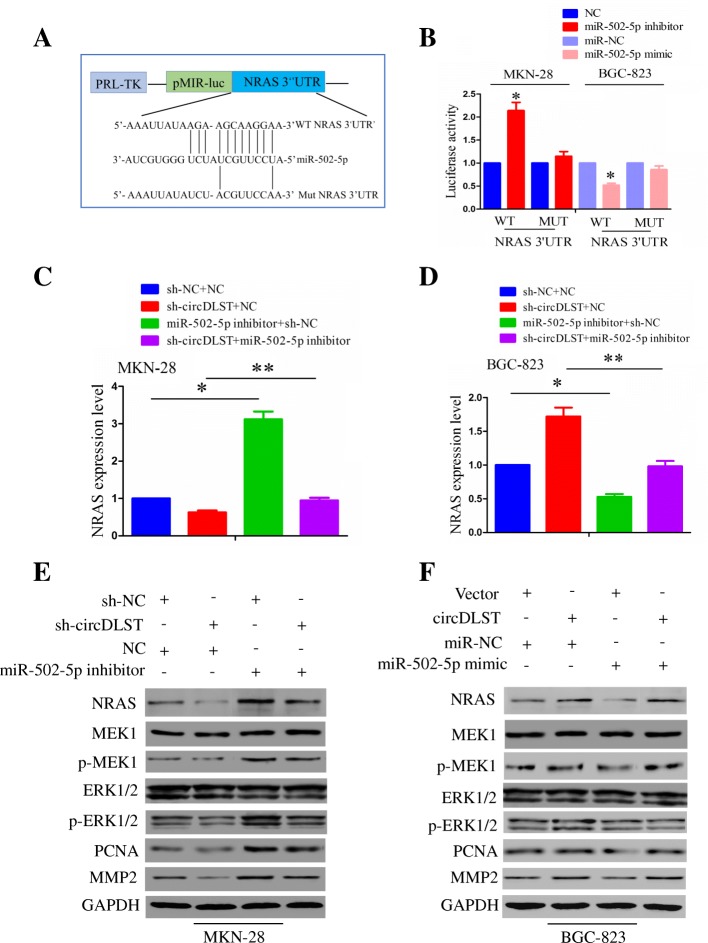
MiR-502-5p reversed circDLST-induced activation of NRAS/MEK1/ERK1/2 signaling in GC cells. **a** Schematic representation of potential binding sites of mi-502-5p with WT or Mut NRAS 3’UTR. **b** The luciferase activity of WT and Mut NRAS 3’UTR after transfection with miR-502-5p inhibitor in MKN-28 cells or miR-502-5p mimic in BGC-823 cells. **c, d** qRT-PCR analysis of NRAS expression levels after the co-transfection of miR-502-5p inhibitor and sh-circDLST in MKN-28 cells or miR-502-5p mimic and circDLST in BGC-823 cells. **e, f** Western blot analysis of the activity of NRAS/MEK1/ERK1/2 signaling and PCNA/MMP2 expression after the co-transfection of miR-502-5p inhibitor and sh-circDLST in MKN-28 cells or miR-502-5p mimic and circDLST in BGC-823 cells. Data are the means ± SEM of three experiments. *P < 0.05; **P < 0.01

NRAS, a target gene of miR-502-5p was screened by using mirPath v.3 and microT_CDS (Additional file 9: Figure S8 and Additional file 1: Table S6). PRL-TK-pMIR-Luc vectors containing the WT or Mut NRAS 3’UTR (Fig. 8a) were co-transfected with miR-502-5p mimic or inhibitor into MKN-28 and BGC-823 cells. We found that, the luciferase activity of WT NRAS 3’UTR was increased by miR-502-5p inhibitor in MKN-28 cells, but decreased by miR-502-5p mimic in BGC-823 cells (*P* < 0.05), while that of Mut NRAS 3’UTR was unaffected by miR-502-5p inhibitor or mimic in MKN-28 or BGC-823 cells as compared with the control group (Fig. 8b). Further investigations showed that, miR-502-5p inhibitor increased NRAS mRNA expression (Fig. 8c), activated NRAS/MEK1/ERK1/2 signaling and increased PCNA/MMP2 expression (Fig. 8e) in MKN-28 cells, but miR-502-5p mimic had an inhibitory effect on them in BGC-823 cells (Fig. 8d, f). Moreover, miR-502-5p inhibitor reversed sh-circDLST induced inhibitory effects on the activated NRAS/MEK1/ ERK1/2 signaling and PCNA/MMP2 expression in MKN-28 cells (Fig. 8c, e), while miR-502-5p mimic attenuated circDLST-induced these effects in BGC-823 cells (Fig. 8d, f).

The transfection efficiency of si-NRAS or NRAS plasmid was assessed by qRT-PCR and Western blot analysis in MKN-28 or BGC-823 cells (*P* < 0.01, Additional file 10: Figure S9A). After the co-transfection of miR-502-5p inhibitor and si-NRAS in MKN-28 cells or miR-502-5p mimic and NRAS in BGC-823 cells, we found that, knockdown of NRAS inhibited the cell proliferation and invasion, and abrogated the tumor-promoting effects of miR-502-5p inhibitor (*P* < 0.05, Additional file 10: Figure S9B, D), while ectopic expression of NRAS showed the opposite effects (*P* < 0.05, Additional file 10: Figure S9C, E).

## Discussion

CircRNAs have been reported as the potential biomarkers for patients with GC. High expression of circPVT1 is identified as an independent prognostic factor of poor survival in patients with GC [21]. Our previous study uncovered circLARP4 as a favorable factor for predicting the survival of patients with GC [20]. Herein, we found that, circDLST expression levels were increased in GC tissues as compared with the adjacent normal tissues, but had no association with the clinicopathological characteristics in patients with GC. Multivariate analysis revealed high expression of circDLST as an independent prognostic factor of poor survival in patients with GC. TNM stage is an important indicator of cancer prognosis, and high expression of circDLST was also associated with poor survival in early or advanced patients with chemotherapy, indicating that, circDLST might be a promising indicator of poor survival in GC patients with or without chemotherapy.

CircRNAs act as oncogenic or tumor suppressive factors in GC. CircFAT1(e2)/circ_100269 repress the proliferation and invasion of GC cells [22, 24], but circPVT1/circSFMBT2 favor their growth [21, 27]. We previously proposed that, circLARP4/circYAP1 repressed the proliferation and invasion of GC cells [20, 28]. Herein, we identified a functional role of circDLST in GC cells and found that, knockdown of circDLST suppressed the cell proliferation, cell invasion and liver metastasis in vitro and in vivo, while re-expression of circDLST had an opposite effect. These results indicated that circDLST might be an oncogenic factor in GC cells.

Mounting evidence shows that, circRNAs act as miRNA sponges to regulate tumor progression [29–31]. circ_100269 sponges miR-630 to inhibit the GC growth [24], while circ-SFMBT2 sponges miR-182 to exert a GC-promoting effect [26]. We previously proposed that, circLARP4/circYAP1 act by sponging miR-424/LATS1 or miR-367p/p27^Kip1^ axis in GC cells [20, 27]. Herein, circDLST was verified to act as a sponge of miR-502-5p in GC cells, which suppresses the growth and cycle progression of colon cancer [32]. We also found that, miR-502-5p was downregulated in GC tissues, inhibited cell proliferation and invasion, and reversed the tumor-promoting effects of circDLST in GC cells. But, duo to the limited sample size of GC patients in TCGA cohort, low expression of miR-502-5p showed no association with the survival and tumor recurrence of GC patients, and it could not be regarded as a diagnostic target of GC. Nevertheless, our results indicated that, circDLST might sponge miR-502-5p to promote the tumorigenesis of GC cells.

**Fig. 9:**
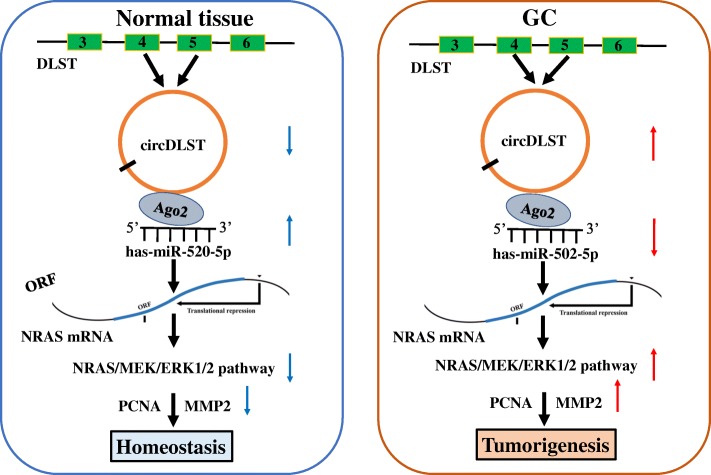
Schematic representation of the proposed mechanism of circDLST in GC cells. circDLST acted as a sponge of miR-502-5p to activate the NRAS/MEK1/ERK1/2 signaling and upregulate PCNA and MMP2 expression, leading to the tumorigenesis and metastasis of GC

We further identified NRAS as a direct target of miR-502-5p in GC cells. NRAS promotes leukemogenesis [33] and NRAS/MEK/ERK signaling is a key therapeutic target in melanoma and acute myelogenous leukemia [34]. Herein, miR-502-5p decreased NRAS expression, caused the inactivation of MEK1/ERK1/2 signaling, and counteracted circDLST-induced activation of NRAS/MEK1/ERK1/2 signaling. PCNA and MMP2 are considered as the proliferation and invasion related markers in GC [35, 36]. MiR-502-5p decreased PCNA and MMP2 expression levels, and reversed circDLST-induced their expression in GC cells. These findings indicated that, circDLST sponged miR-502-5p to activate the NRAS/MEK1/ERK1/2 signaling and thereby upregulated PCNA and MMP2 expression, leading to the tumorigenesis of GC (Fig. 9).

## Conclusion

Taken together, our findings demonstrated that, circDLST promoted the tumorigenesis and metastasis of GC cells by sponging miR-502-5p and activating the NRAS/MEK1/ERK1/2 signaling, and high expression of circDLST acted as an independent prognostic factor of poor survival in GC patients.

## Additional fileS

Additional file 1: **Table S1.** The primer sequences. **Table S2.** Correlation of circDLST expression with clinicopathologic features of GC patients. **Table S3.** Univariate and multivariate Cox regression analysis of the association of circDLST with poor survival in GC patients. **Table S4.** Ago2 occupancy in the region of circDLST. **Table S5.** RIP-miRNA-seq identification of the upregulated miRNAs. **Table S6.** Top target genes of has-miR-502-5p. (DOCX 34 kb)

Additional file 2: **Figure S1.** The cutoff value of circDLST divided the GC patients into circDLST high and low expression groups. (PDF 51 kb)

Additional file 3: **Figure S2.** FISH analysis of the association of circDLST expression levels with the clinicopathological characteristics of GC patients. (PDF 209 kb)

Additional file 4: **Figure S3.** Kaplan Meier analysis of the association of chemotherapy or non-chemotherapy with overall survival in patients with GC. (PDF 59 kb)

Additional file 5: **Figure S4.** Schematic representation of potential binding sites of miRNAs with WT or MUT circDLST. (PDF 228 kb)

Additional file 6: **Figure S5.** TCGA analysis of the expression levels of miR-193b-5p, miR-542-3p, miR-362-5p and miR-203a-5p in paired and unpaired GC tissues. (PDF 229 kb)

Additional file 7: **Figure S6.** TCGA analysis of the association of high or low miR-502-5p expression with the overall survival and tumor recurrence of GC patients. (PDF 343 kb)

Additional file 8: **Figure S7. **qRT-PCR analysis of the expression levels of miR-502-5p and its correlation with circDLST in GC cell lines. (PDF 49 kb)

Additional file 9: **Figure S8.** Schematic representation of the involvement of NRAS in MEK/ERK signaling pathway. (PDF 1238 kb)

Additional file 10: **Figure S9.** NRAS reversed the tumor-suppressive effects of miR-502-5p in GC cells. (A) qRT-PCR and Western blot analysis of the transfection efficiency of si-NRAS or NRAS plasmid in MKN-28 or BGC-823 cells. (B-E) MTT and Transwell analysis of the cell viability and invasive potential after the co-transfection of miR-502-5p inhibitor and si-NRAS in MKN-28 cells or miR-502-5p mimic and NRAS in BGC-823 cells. Bar scale: 125 μm. Data are the means ± SEM of three experiments. *P < 0.05; **P < 0.01. (PDF 565 kb)

## Abbreviations

CircRNA: Circular RNA
ERK: Extracellular regulated MAP kinase
FBS: Fetal bovine serum
FISH: Fluorescence in situ hybridization
GC: Gastric cancer
IOD: Immunofluorescence accumulation optical density
MiRNA: MicroRNA
NcRNAs: Non-coding RNAs
qRT-PCR: Quantitative real-time PCR
RIP: RNA immunoprecipitation
TCGA: The Cancer Genome Atlas
TMA: Tissue microarray
TNM: Tumor-Node-Metastasis
