# Correction to: CircDLST promotes the tumorigenesis and metastasis of gastric cancer by sponging miR-502-5p and activating the NRAS/MEK1/ERK1/2 signaling

**DOI:** 10.1186/s12943-020-01243-0

**Published:** 2020-08-12

**Authors:** Jing Zhang, Lidan Hou, Rui Liang, Xiaoyu Chen, Rui Zhang, Wei Chen, Jinshui Zhu

**Affiliations:** 1 Department of Gastroenterology, Shanghai Jiao Tong University Affiliated Sixth People’s Hospital, No. 600 Yishan Road, Shanghai, 200233 China; 2 Department of Gastroenterology, Shanghai Ninth People’s Hospital, Shanghai Jiao Tong University School of Medicine, Shanghai, China

**Correction to: Mol Cancer 18, 80 (2019)**

**https://doi.org/10.1186/s12943-019-1015-1**

Following publication of the original article [1], the authors found an error in Fig. 7d, e. The correct figure is shown below.

**Fig. 7:**
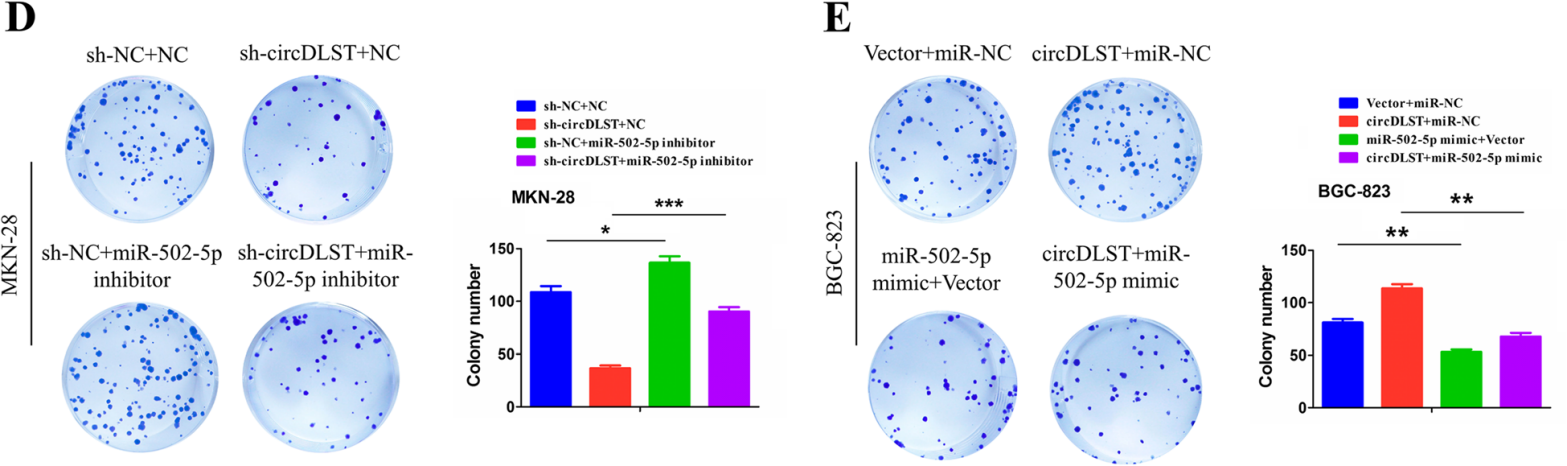
**d**, **e** Colony formation analysis of the colony number after the co-transfection of miR-502-5p inhibitor and sh-circDLST in MKN-28 cells or miR-502-5p mimic and circDLST in BGC-823 cells. Data are the means ± SEM of three experiments. *P < 0.05; **P < 0.01; ***P < 0.001

## References

[1] Zhang J, Hou L, Liang R, Chen X, Zhang R, Chen W, Zhu J (2019). CircDLST promotes the tumorigenesis and metastasis of gastric cancer by sponging miR-502-5p and activating the NRAS/MEK1/ERK1/2 signaling. Mol Cancer.

