# Supplementary material for: CircDLST promotes the tumorigenesis and metastasis of gastric cancer by sponging miR-502-5p and activating the NRAS/MEK1/ERK1/2 signaling
Source: Mol Cancer. 2019 Apr 5;18:80. doi: 10.1186/s12943-019-1015-1 (PMC6449953; doi:10.1186/s12943-019-1015-1)
Supplement: Supplementary file 1 — Table S1. The primer sequences. Table S2. Correlation of circDLST expression with clinicopathologic features of GC patients. Table S3. Univariate and multivariate Cox regression analysis of the association of circDLST with poor survival in GC patients. Table S4. Ago2 occupancy in the region of circDLST. Table S5. RIP-miRNA-seq identification of the upregulated miRNAs. Table S6. Top target genes of has-miR-502-5p. (DOCX 34 kb) [file 12943_2019_1015_MOESM1_ESM.docx]

Additional file 1

Table S1 The primer sequences

| **Gene name** | **Primer sequence** | Annealing temperature (℃) | Product length (bp) |
| --- | --- | --- | --- |
| β-actin（Human） | F:5' GTGGCCGAGGACTTTGATTG3'  R:5’CCTGTAACAACGCATCTCATATT3’ | 60 | 73 |
| circDLST | F:5’TACAGTCAAAACCCCAGCGT3’  R:5’GTTGTTAATGCTTTCTCCCACC3’ | 60 | 72 |
| miR-502-5p | F: 5’ATCCTTGCTATCTGGGTGCTA3’ | 60 |  |
| NRAS | F: 5’TGAGAGACCAATACATGAGGACA3’  R:5’CCCTGTAGAGGTTAATATCCGCA3’ | 60 | 93 |

Table S2 Correlation of circDLST expression with clinicopathologic

features of GC patients

| clinicopathologic features | Cases  (n) | circDLST expression | | *P* |
| --- | --- | --- | --- | --- |
|  | 71 | Low  n=58 | High n=13 |  |
| ***Age*** |  |  |  |  |
| ≥60 | 32 | 25 | 7 |  |
| <60 | 39 | 33 | 6 | 0.485 |
| ***Gender*** |  |  |  |  |
| Female | 26 | 23 | 3 |  |
| Male | 45 | 35 | 10 | 0.266 |
| ***Tumor size (cm)*** |  |  |  |  |
| <3.5 | 31 | 24 | 7 |  |
| ≥3.5 | 40 | 34 | 6 | 0.363 |
| ***Pathological stage*** |  |  |  |  |
| I+II | 32 | 28 | 4 |  |
| III | 39 | 30 | 9 | 0.255 |
| ***Tumor size*** |  |  |  |  |
| Gastric antrum | 34 | 26 | 8 |  |
| Lesser curvature | 16 | 14 | 2 |  |
| Gastric body | 7 | 7 | 0 |  |
| Gastric cardia and other | 14 | 11 | 3 | 0.457 |
| ***TNM staging*** |  |  |  |  |
| 1+2 | 34 | 30 | 4 |  |
| 3+4 | 37 | 28 | 9 | 0.175 |
| ***Lymph node metastasis*** |  |  |  |  |
| Negative | 24 | 21 | 3 |  |
| Positive | 47 | 37 | 10 | 0.369 |
| ***Chemotherapy*** |  |  |  |  |
| Negative | 11 | 11 | 0 |  |
| Positive | 60 | 47 | 13 | 0.090 |

Table S3 Univariate and multivariate Cox regression analysis of

the association of circDLST with poor survival in GC patients

| Parameter | Univariate *P* |  | Multivariate analysis | | |
| --- | --- | --- | --- | --- | --- |
|  |  |  | *P* | HR | 95%CI |
| Age (≥60 vs. <60 years) | 0.725 |  | NA |  |  |
| Gender (Male vs. Female) | 0.024 |  | 0.023 | 2.199 | 1.113-4.341 |
| Tumor size (≥3.5 vs. <3.5 cm) | 0.113 |  | NA |  |  |
| Pathological staging (I/II vs. III) | 0.496 |  | NA |  |  |
| Tumor site (Gastric antrum vs lesser curvature  vs gastric body vs gastric cardia and other) | 0.094 |  | NA |  |  |
| TNM staging (1/2 vs. 3/4) | 0.959 |  | NA |  |  |
| Lymph node metastasis (Positive vs. Negative) | 0.627 |  | NA |  |  |
| Chemotherapy (Positive vs. Negative) | 0.909 |  | NA |  |  |
| circDLST expression (High vs. low) | <0.0001 |  | <0.0001 | 4.354 | 1.997-9.492 |

NA: not analyzed

Table S4 Ago2 occupancy in the region of circDLST

| circRNA | Tag Name | % Identity | Alignment Length | Tag Start | Tag End | circRNA Start | circRNA End | Upstream/Downstream |
| --- | --- | --- | --- | --- | --- | --- | --- | --- |
| hsa_circ_0032627 | HHFCT_48492_cluster-6503_7_8_26 | 100.00 | 25 | 2 | 26 | -1000 | -976 | Upstream |

Table S5 RIP-miRNA-seq identification of the upregulated miRNAs

| **mature-miRNA** | **Fold Change** | **Regulation** | **MKN45-RIP** | **MKN45-IgG** |
| --- | --- | --- | --- | --- |
| hsa-let-7f-5p | 1.508705628 | up | 22355 | 14817 |
| hsa-let-7f-5p | 1.508705628 | up | 22355 | 14817 |
| hsa-let-7g-5p | 2.246445498 | up | 7109 | 3164 |
| hsa-let-7i-5p | 1.503484717 | up | 99017 | 65858 |
| hsa-miR-148b-3p | 1.771276596 | up | 332 | 187 |
| hsa-miR-502-5p | 2.105714286 | up | 736 | 349 |
| hsa-miR-21-5p | 1.554809453 | up | 17828 | 11466 |
| hsa-miR-212-5p | 2 | up | 1 | 1 |
| hsa-miR-25-5p | 2 | up | 1 | 1 |
| hsa-miR-30c-2-3p | 1.666666667 | up | 4 | 2 |
| hsa-miR-378a-5p | 3 | up | 2 | 1 |
| hsa-miR-novel-chr5_23809 | 1.611764706 | up | 273 | 169 |
| hsa-miR-novel-chr8_29428 | 4.75 | up | 37 | 7 |
| hsa-miR-novel-chr9_30375 | 2.333333333 | up | 6 | 2 |

Table S6 Top target genes of has-miR-502-5p

| Transcript Id | Target Gene Id (name) | Mirna Name | miTG score |
| --- | --- | --- | --- |
| ENST00000319386 | ENSG00000180347 (CCDC129) | hsa-miR-502-5p | 0.99804 |
| ENST00000334482 | ENSG00000167815 (PRDX2) | hsa-miR-502-5p | 0.997849 |
| ENST00000532592 | ENSG00000170743 (SYT9) | hsa-miR-502-5p | 0.996385 |
| ENST00000584709 | ENSG00000141293 (SKAP1) | hsa-miR-502-5p | 0.9957 |
| ENST00000311481 | ENSG00000174903 (RAB1B) | hsa-miR-502-5p | 0.99388 |
| ENST00000592191 | ENSG00000006125 (AP2B1) | hsa-miR-502-5p | 0.993843 |
| ENST00000369831 | ENSG00000213366 (GSTM2) | hsa-miR-502-5p | 0.992935 |
| ENST00000503468 | ENSG00000069869 (NEDD4) | hsa-miR-502-5p | 0.992331 |
| ENST00000333129 | ENSG00000182263 (FIGN) | hsa-miR-502-5p | 0.986113 |
| ENST00000393651 | ENSG00000135250 (SRPK2) | hsa-miR-502-5p | 0.984972 |
| ENST00000246199 | ENSG00000125975 (C20orf173) | hsa-miR-502-5p | 0.984247 |
| ENST00000415960 | ENSG00000121446 (RGSL1) | hsa-miR-502-5p | 0.983253 |
| ENST00000367602 | ENSG00000116260 (QSOX1) | hsa-miR-502-5p | 0.982403 |
| ENST00000422974 | ENSG00000255073 (ZFP91-CNTF) | hsa-miR-502-5p | 0.98149 |
| ENST00000499247 | ENSG00000141232 (TOB1) | hsa-miR-502-5p | 0.980893 |
| ENST00000265866 | ENSG00000096746 (HNRNPH3) | hsa-miR-502-5p | 0.980813 |
| ENST00000380491 | ENSG00000177627 (C12orf54) | hsa-miR-502-5p | 0.980809 |
| ENST00000295872 | ENSG00000163611 (SPICE1) | hsa-miR-502-5p | 0.979075 |
| ENST00000399044 | ENSG00000214819 (CDRT15L2) | hsa-miR-502-5p | 0.979015 |
| ENST00000435320 | ENSG00000115896 (PLCL1) | hsa-miR-502-5p | 0.977199 |
| ENST00000426902 | ENSG00000105647 (PIK3R2) | hsa-miR-502-5p | 0.976511 |
| ENST00000536061 | ENSG00000248835 (AL357673.1) | hsa-miR-502-5p | 0.97569 |
| ENST00000369535 | ENSG00000213281 (NRAS) | hsa-miR-502-5p | 0.974651 |
| ENST00000545990 | ENSG00000111254 (AKAP3) | hsa-miR-502-5p | 0.973848 |
| ENST00000373644 | ENSG00000138336 (TET1) | hsa-miR-502-5p | 0.973728 |
| ENST00000261349 | ENSG00000070018 (LRP6) | hsa-miR-502-5p | 0.972141 |
| ENST00000382376 | ENSG00000186458 (DEFB132) | hsa-miR-502-5p | 0.970921 |
| ENST00000441049 | ENSG00000188010 (MORN2) | hsa-miR-502-5p | 0.970132 |
| ENST00000418342 | ENSG00000169641 (LUZP1) | hsa-miR-502-5p | 0.969822 |
| ENST00000455903 | ENSG00000155636 (RBM45) | hsa-miR-502-5p | 0.967559 |
| ENST00000333279 | ENSG00000183831 (ANKRD45) | hsa-miR-502-5p | 0.966785 |
| ENST00000374237 | ENSG00000183675 (PTPN20B) | hsa-miR-502-5p | 0.964953 |
| ENST00000369458 | ENSG00000134258 (VTCN1) | hsa-miR-502-5p | 0.964771 |
| ENST00000421012 | ENSG00000014641 (MDH1) | hsa-miR-502-5p | 0.964237 |
| ENST00000395894 | ENSG00000187607 (ZNF286A) | hsa-miR-502-5p | 0.964169 |
| ENST00000587228 | ENSG00000186566 (GPATCH8) | hsa-miR-502-5p | 0.963324 |
| ENST00000269197 | ENSG00000141431 (ASXL3) | hsa-miR-502-5p | 0.960754 |
| ENST00000412731 | ENSG00000128708 (HAT1) | hsa-miR-502-5p | 0.957402 |
| ENST00000265715 | ENSG00000091137 (SLC26A4) | hsa-miR-502-5p | 0.956122 |
| ENST00000259254 | ENSG00000136732 (GYPC) | hsa-miR-502-5p | 0.955729 |
| ENST00000377507 | ENSG00000049249 (TNFRSF9) | hsa-miR-502-5p | 0.954656 |
| ENST00000409638 | ENSG00000115661 (STK16) | hsa-miR-502-5p | 0.954209 |
| ENST00000586592 | ENSG00000152234 (ATP5A1) | hsa-miR-502-5p | 0.953992 |
| ENST00000488381 | ENSG00000111653 (ING4) | hsa-miR-502-5p | 0.953894 |
| ENST00000293780 | ENSG00000108556 (CHRNE) | hsa-miR-502-5p | 0.952451 |
| ENST00000376185 | ENSG00000254870 (ATP6V1G2-DDX39B) | hsa-miR-502-5p | 0.952191 |
