# Supplementary figures and images for: CircDLST promotes the tumorigenesis and metastasis of gastric cancer by sponging miR-502-5p and activating the NRAS/MEK1/ERK1/2 signaling
Source: Mol Cancer. 2019 Apr 5;18:80. doi: 10.1186/s12943-019-1015-1 (PMC6449953; doi:10.1186/s12943-019-1015-1)

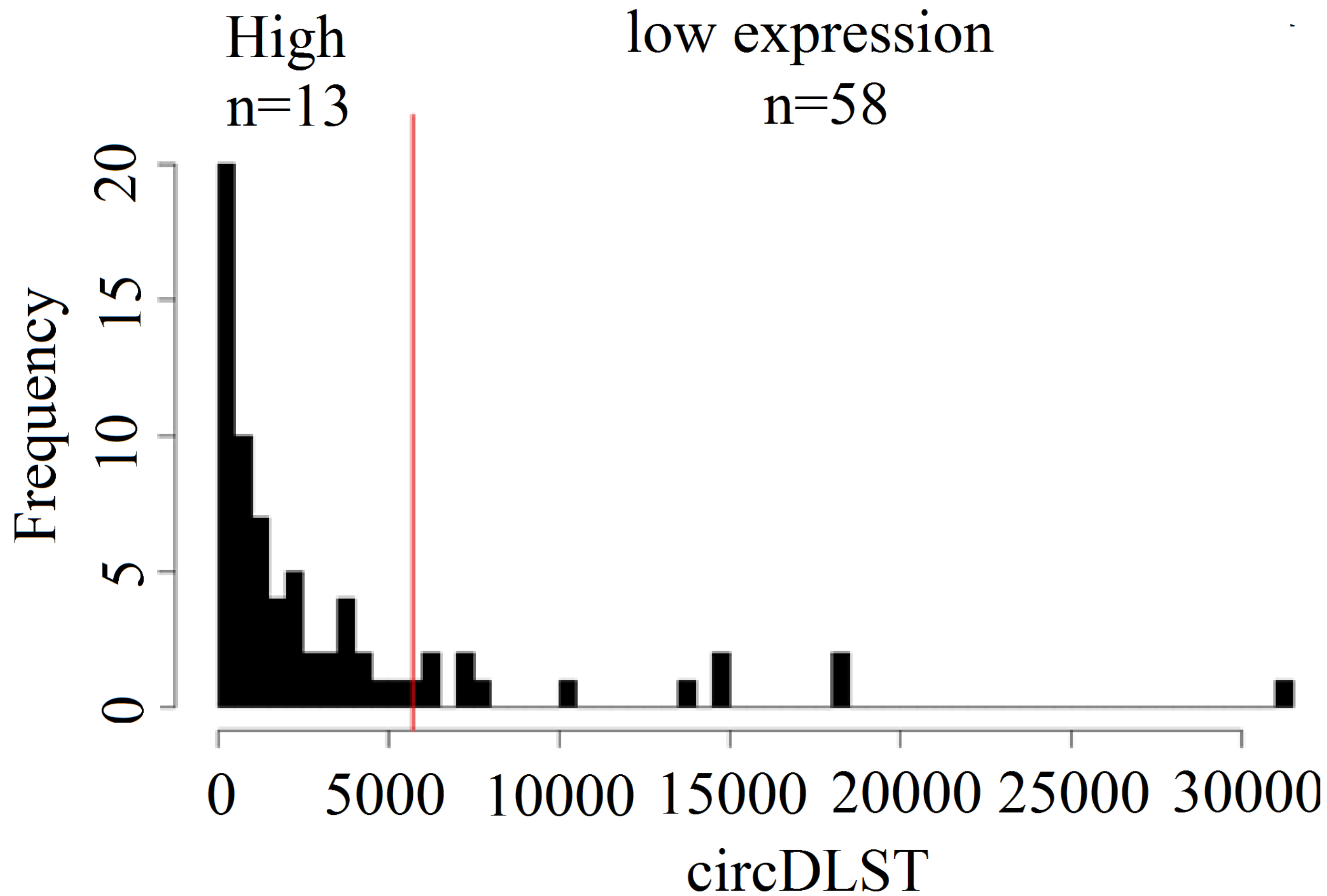

Supplement: Supplementary file 2 — Figure S1. The cutoff value of circDLST divided the GC patients into circDLST high and low expression groups. (PDF 51 kb) [file 12943_2019_1015_MOESM2_ESM.pdf]

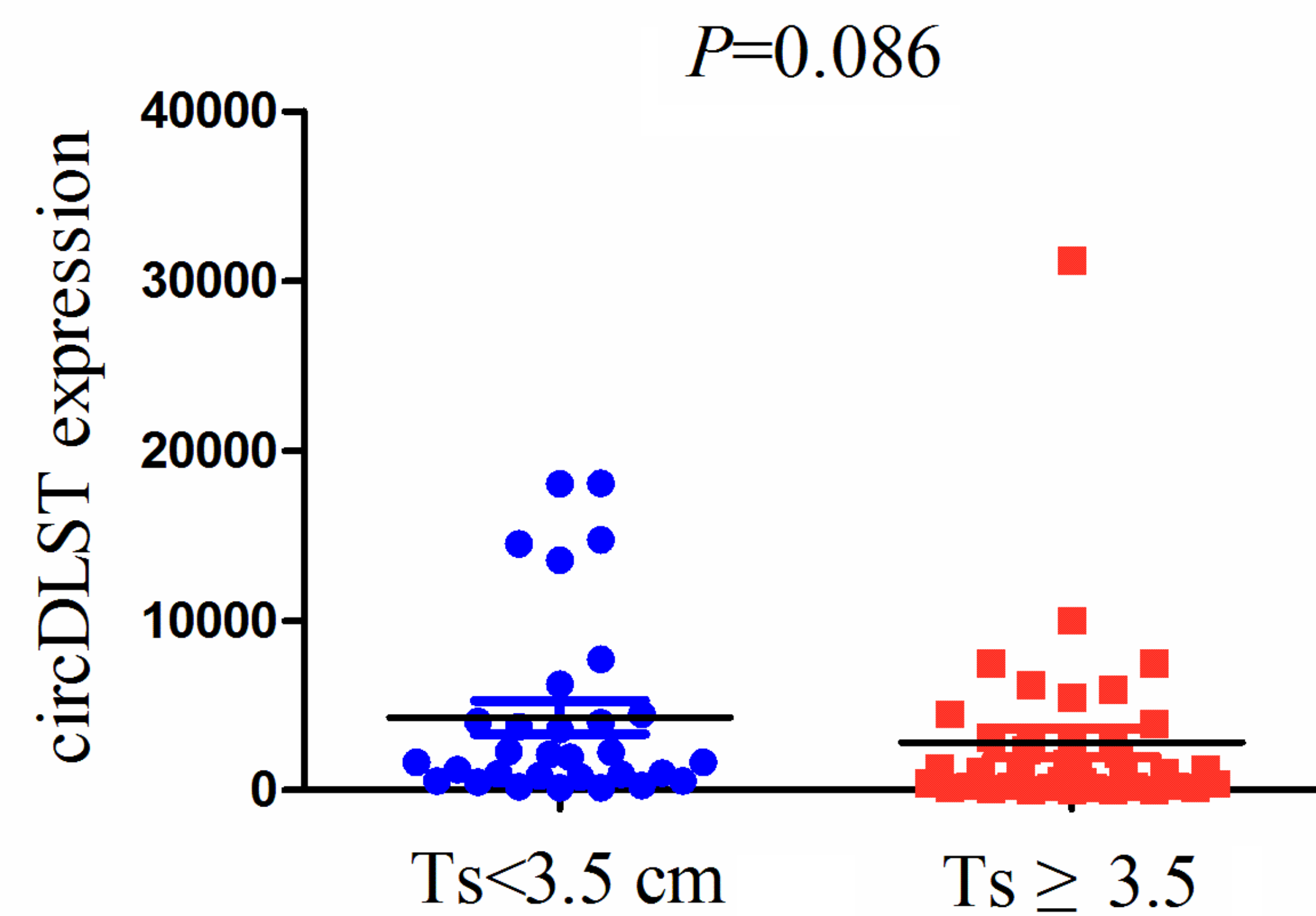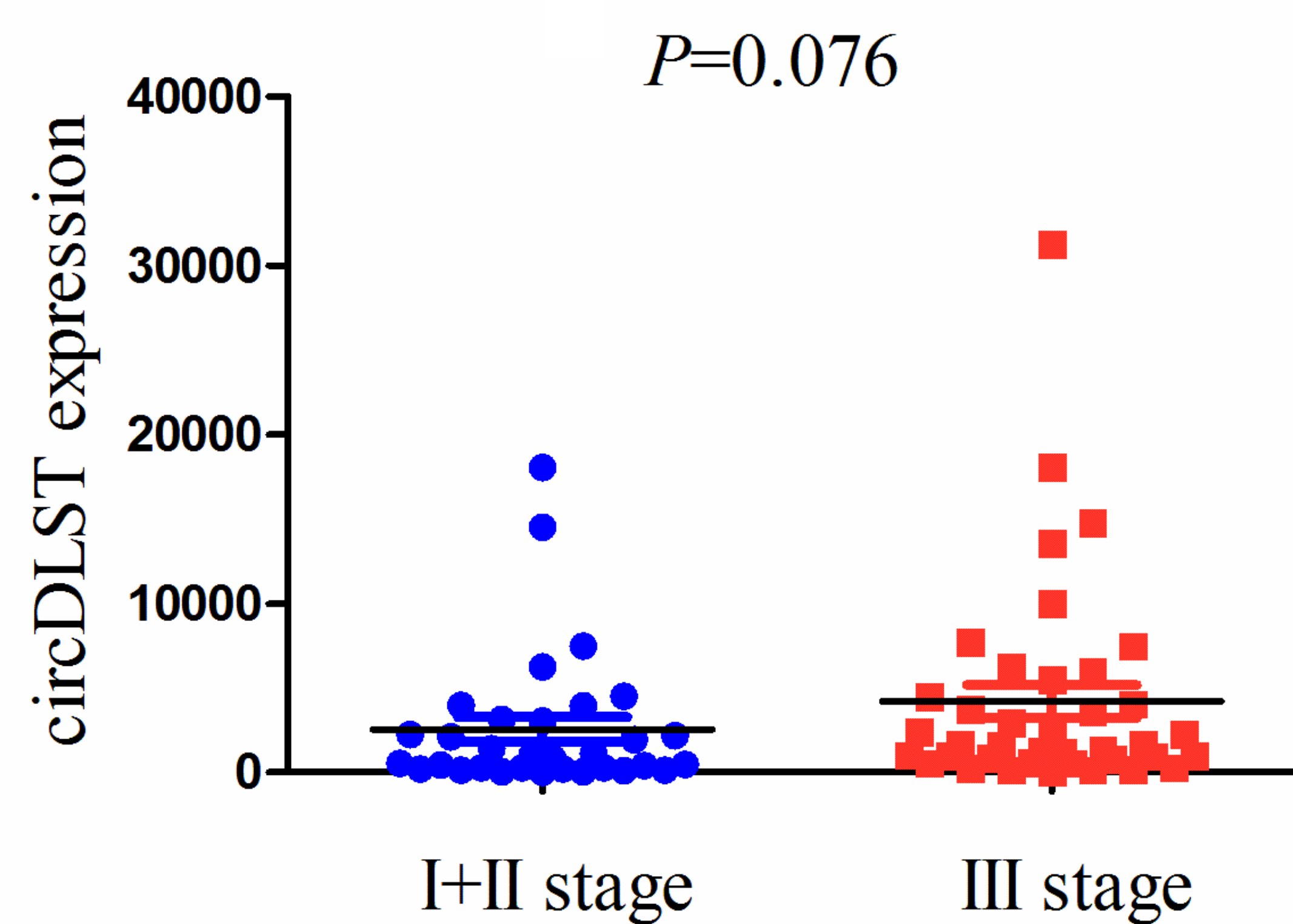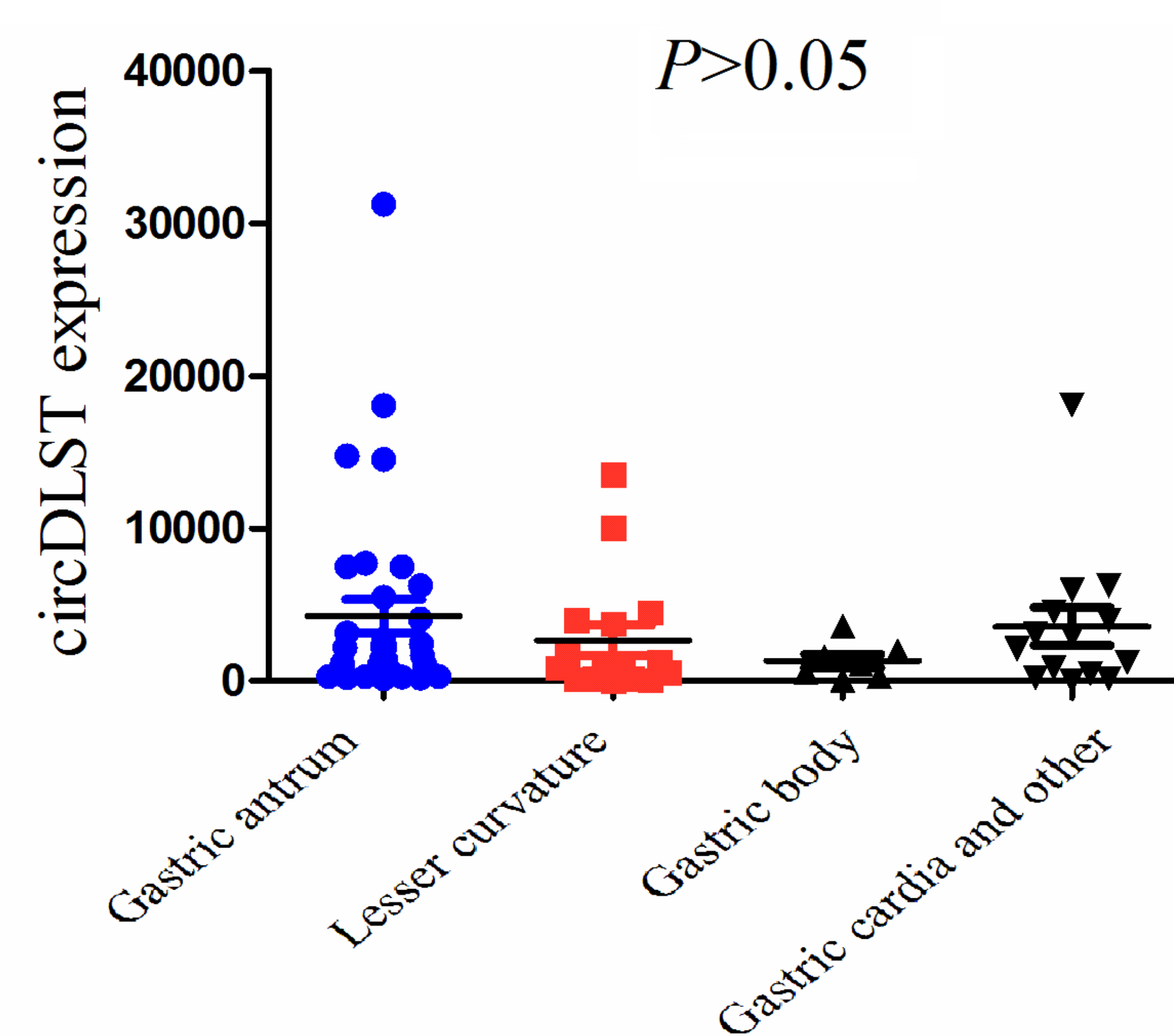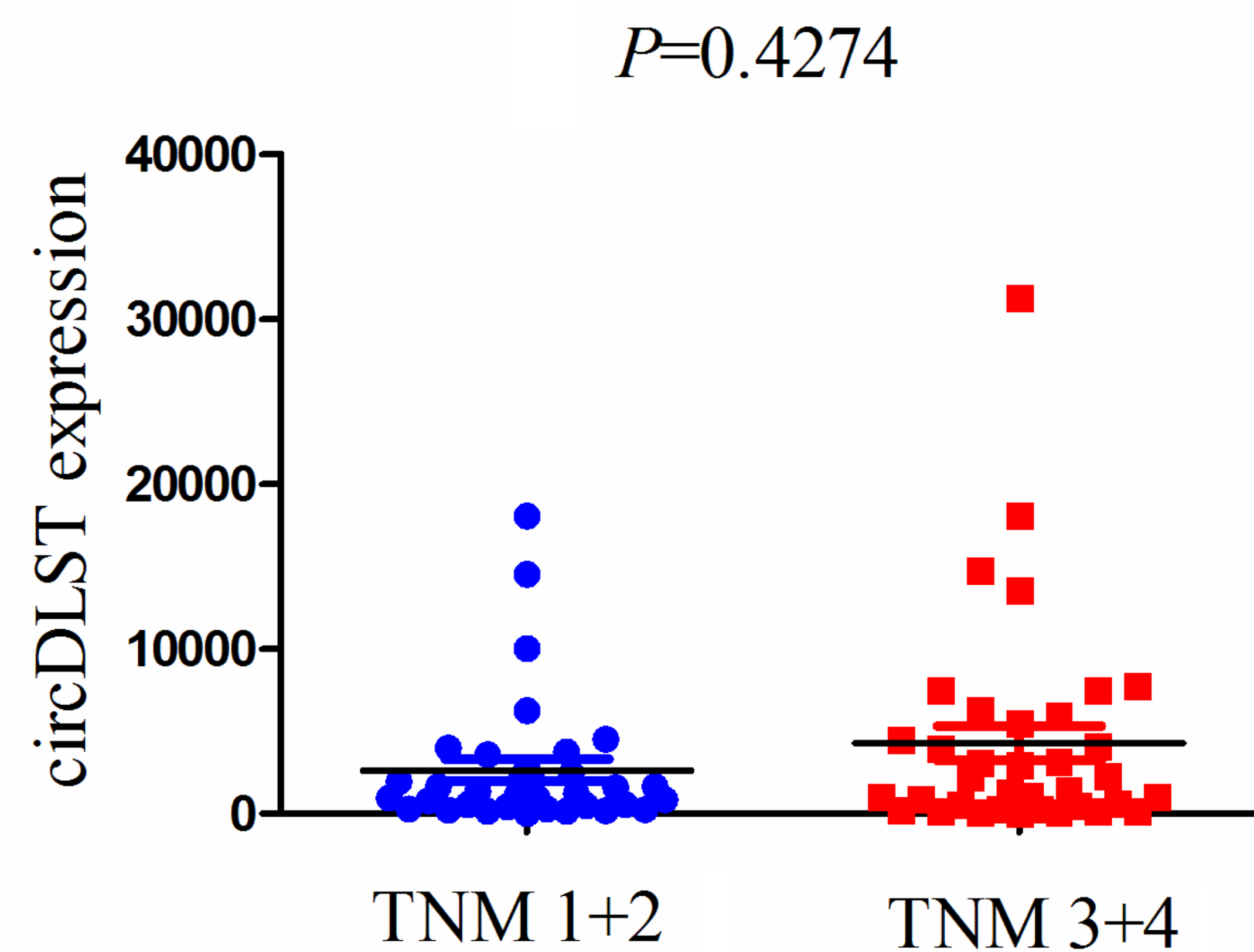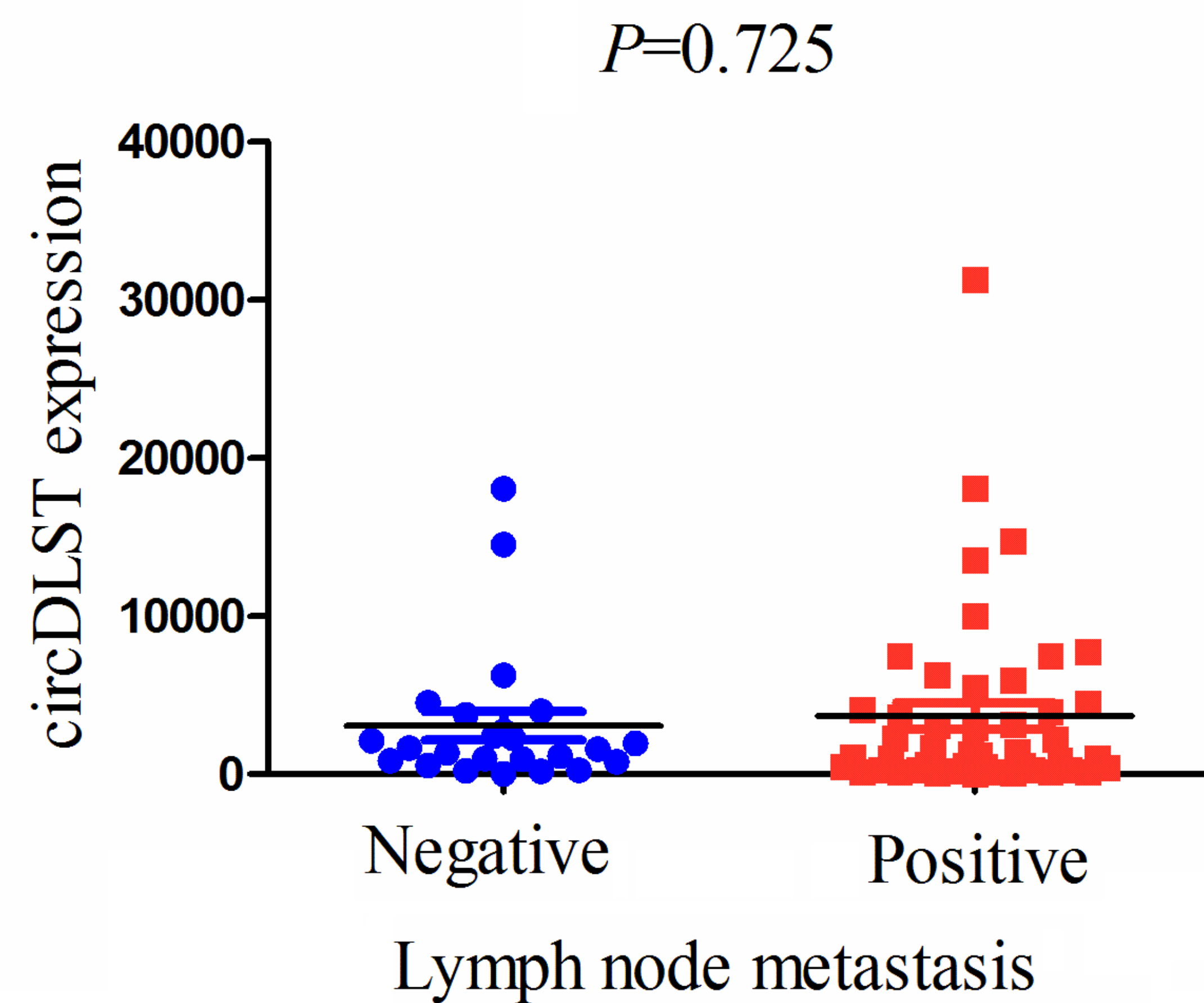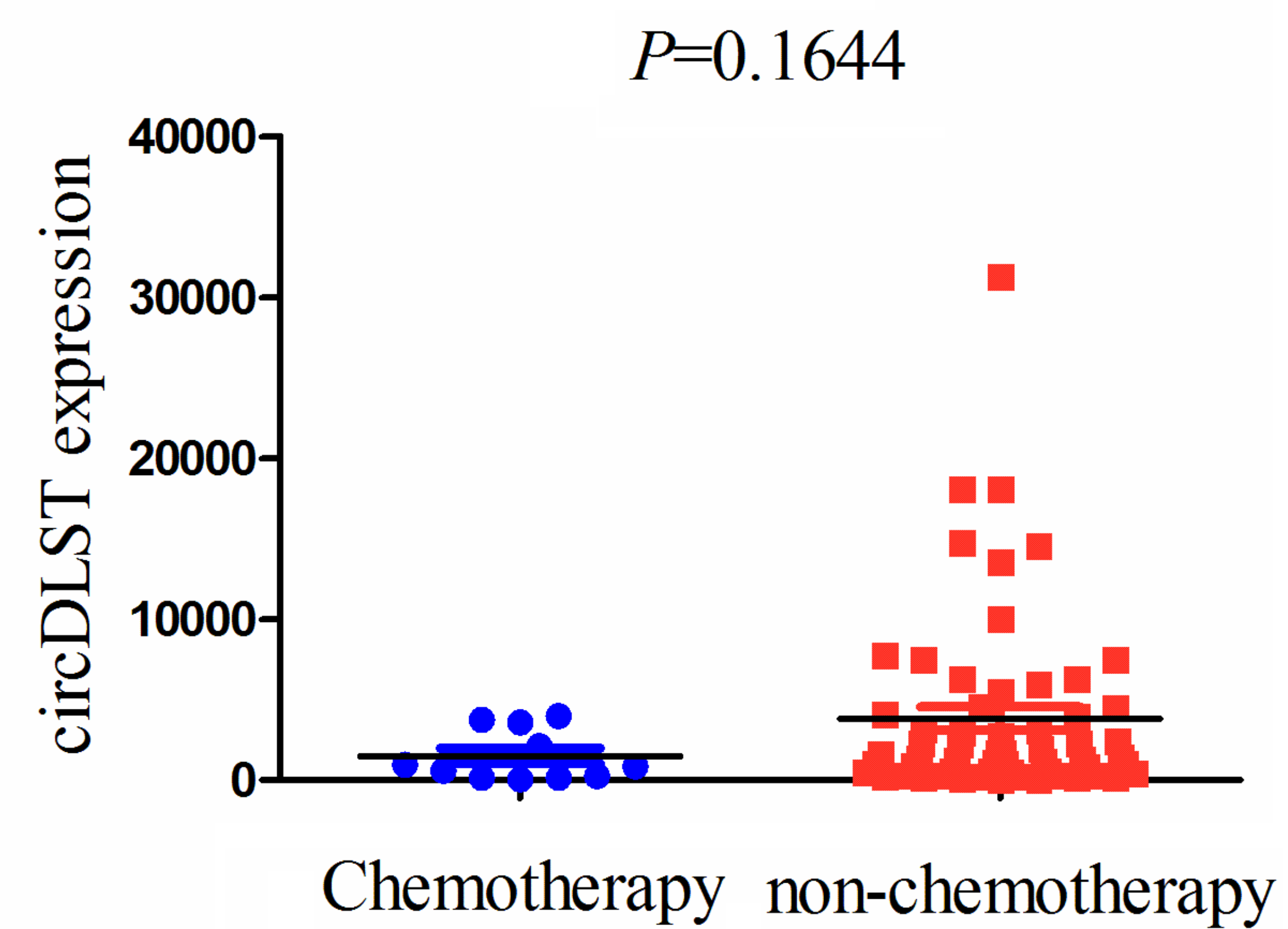

Supplement: Supplementary file 3 — Figure S2. FISH analysis of the association of circDLST expression levels with the clinicopathological characteristics of GC patients. (PDF 209 kb) [file 12943_2019_1015_MOESM3_ESM.pdf]

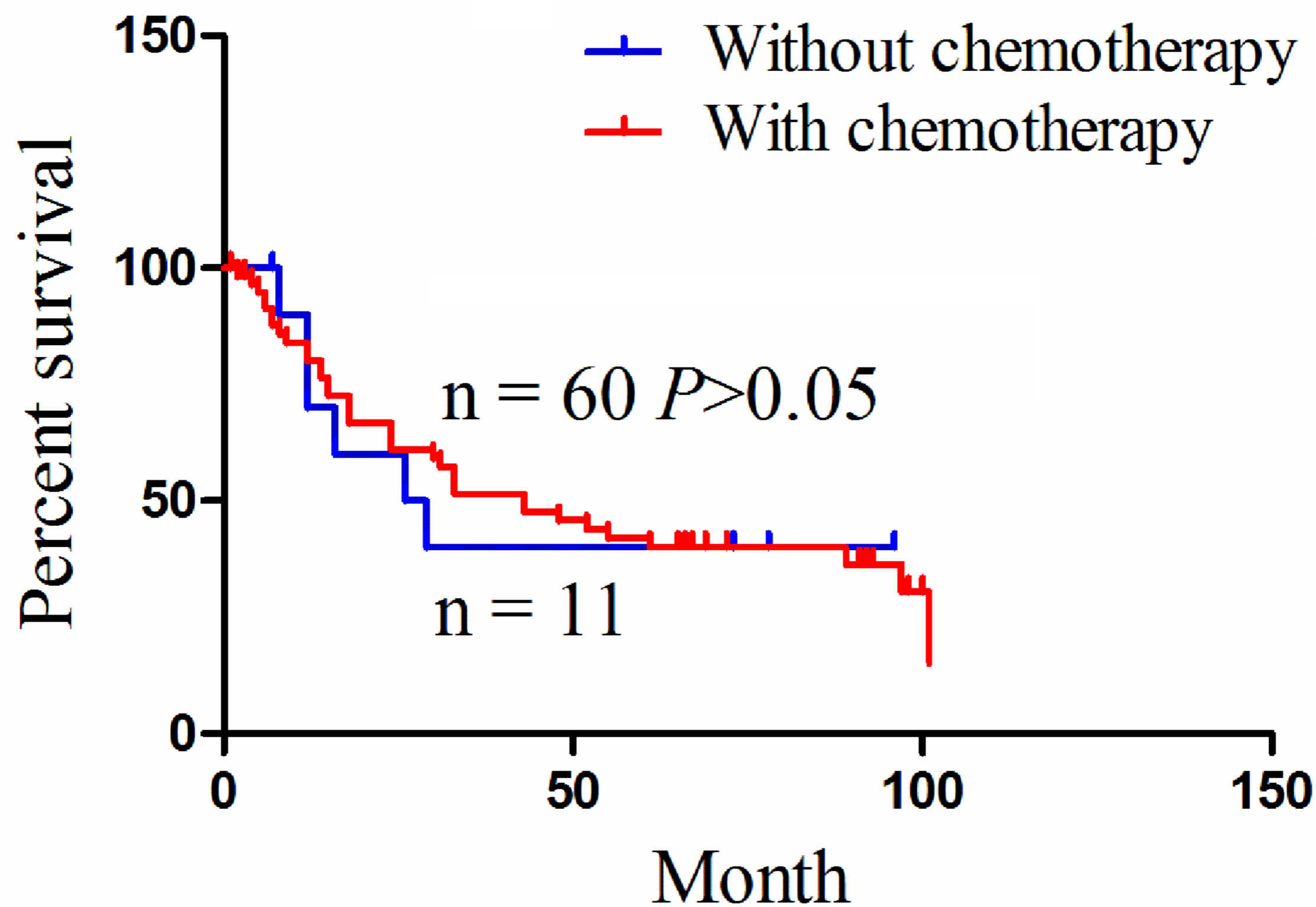

Supplement: Supplementary file 4 — Figure S3. Kaplan Meier analysis of the association of chemotherapy or non-chemotherapy with overall survival in patients with GC. (PDF 59 kb) [file 12943_2019_1015_MOESM4_ESM.pdf]

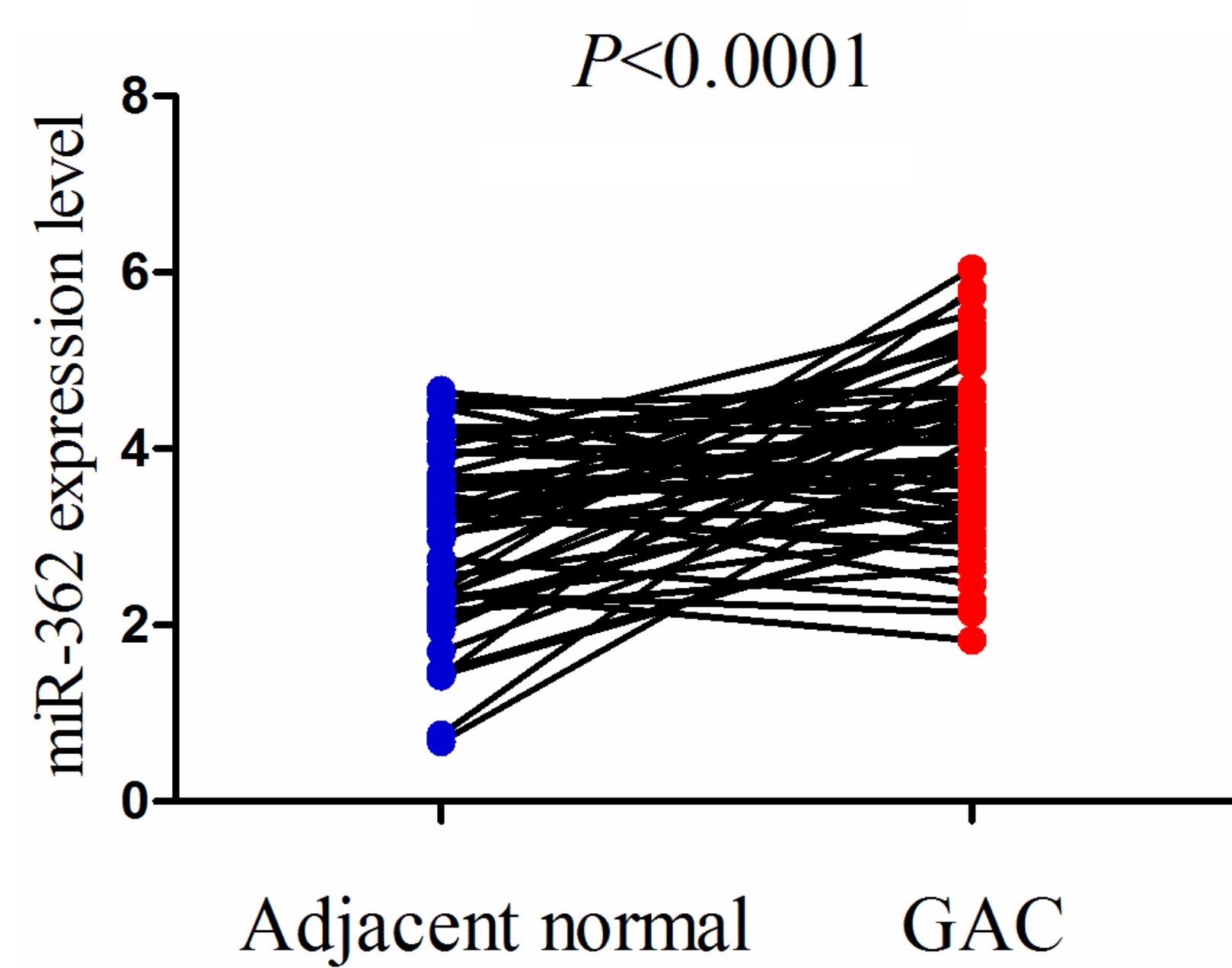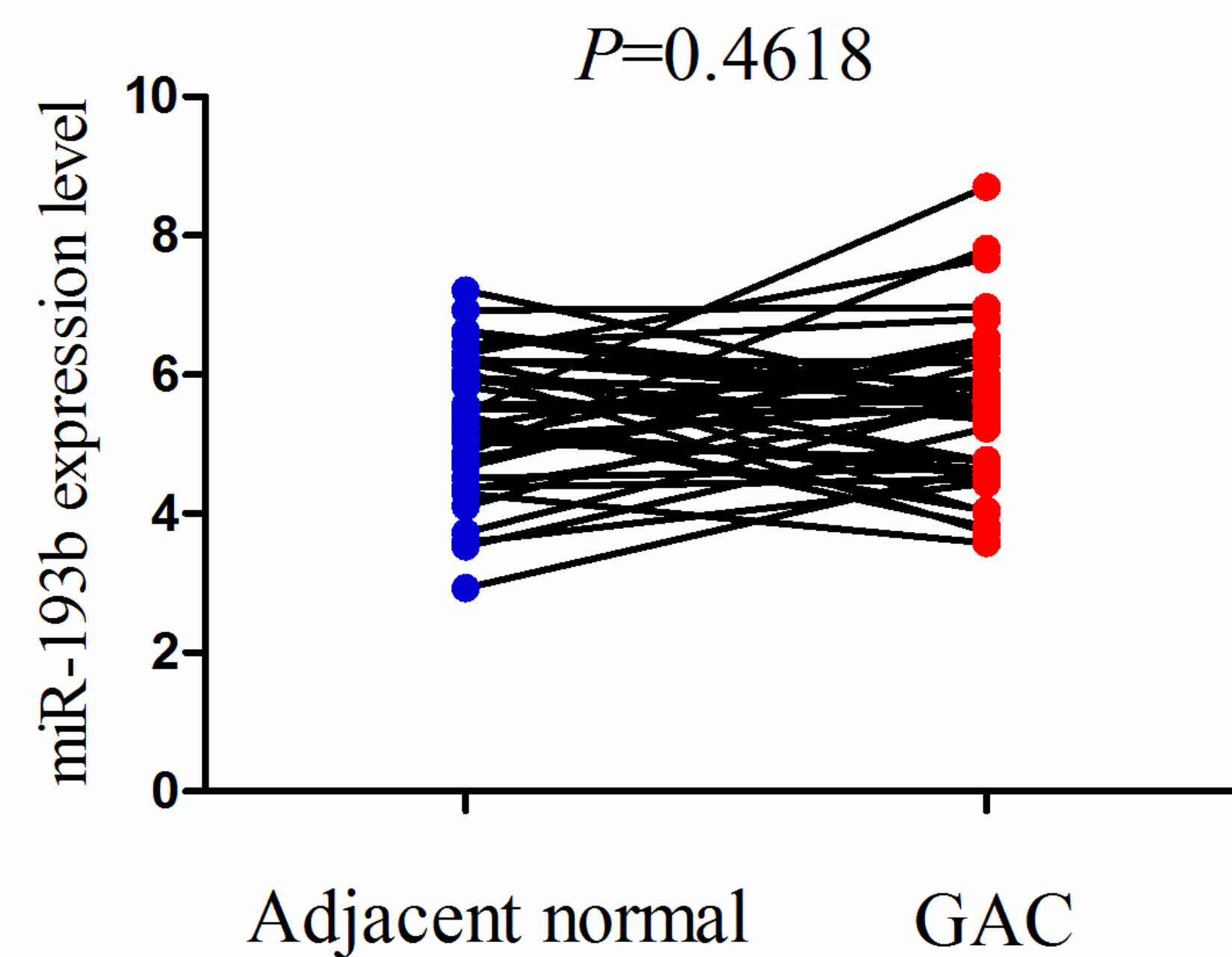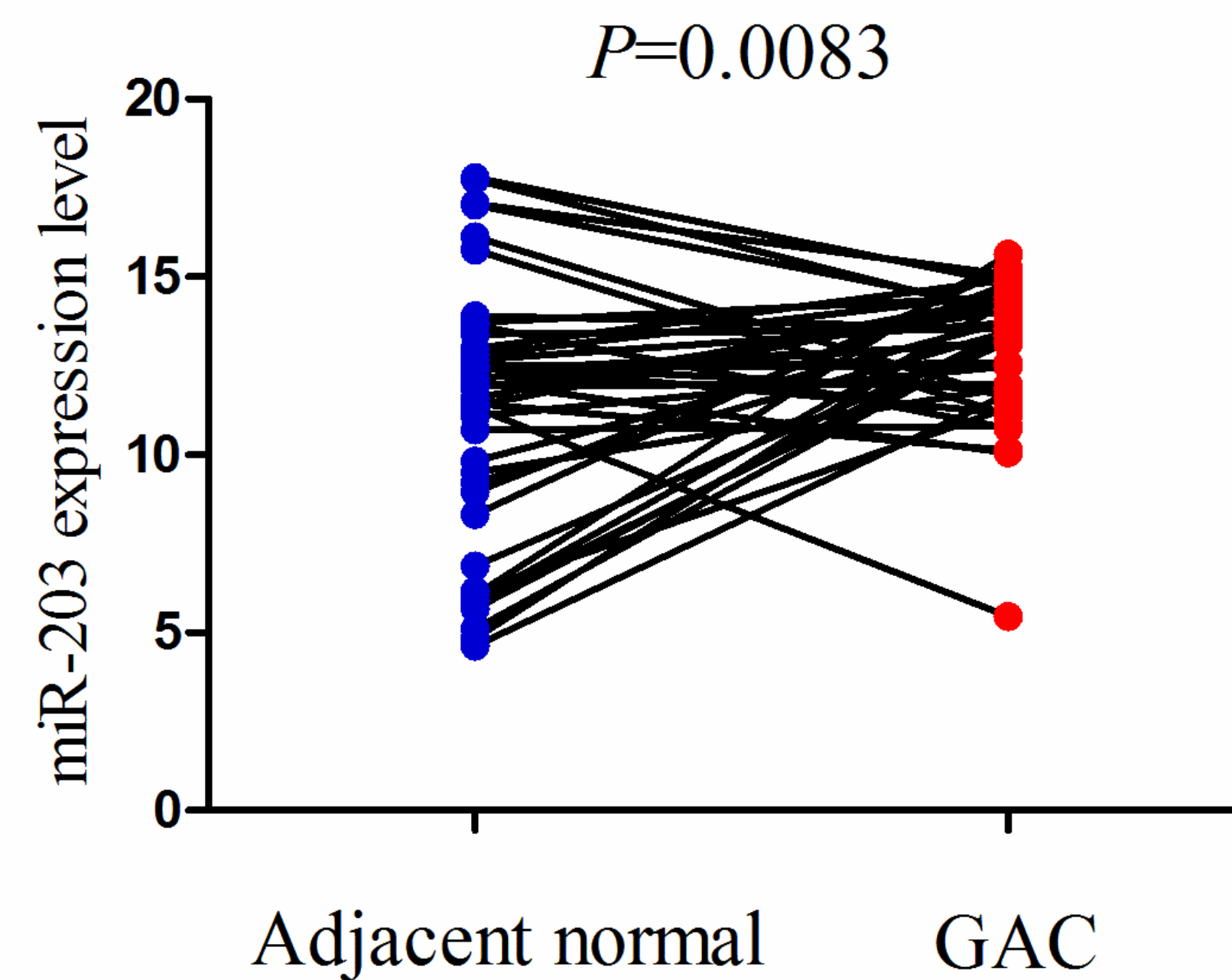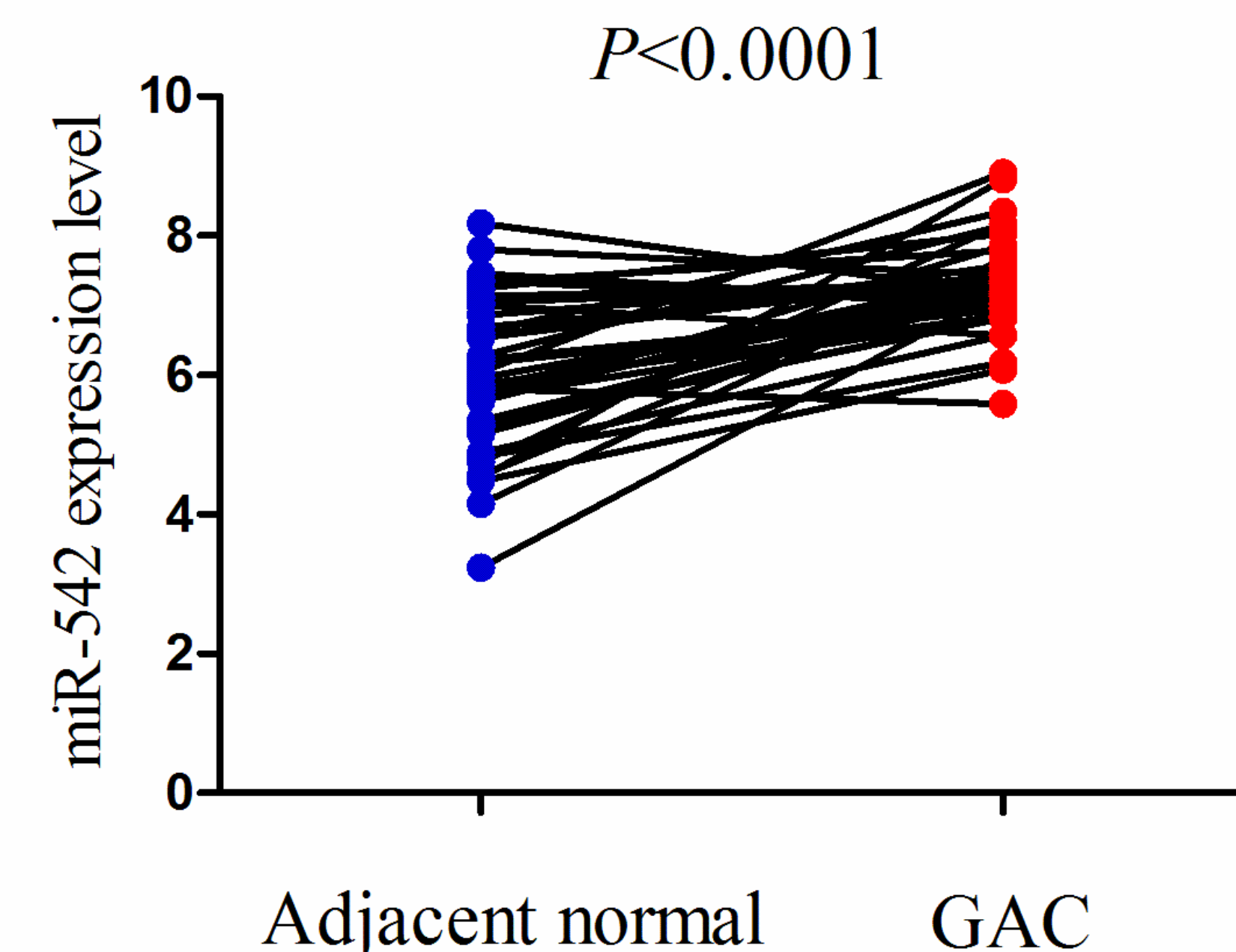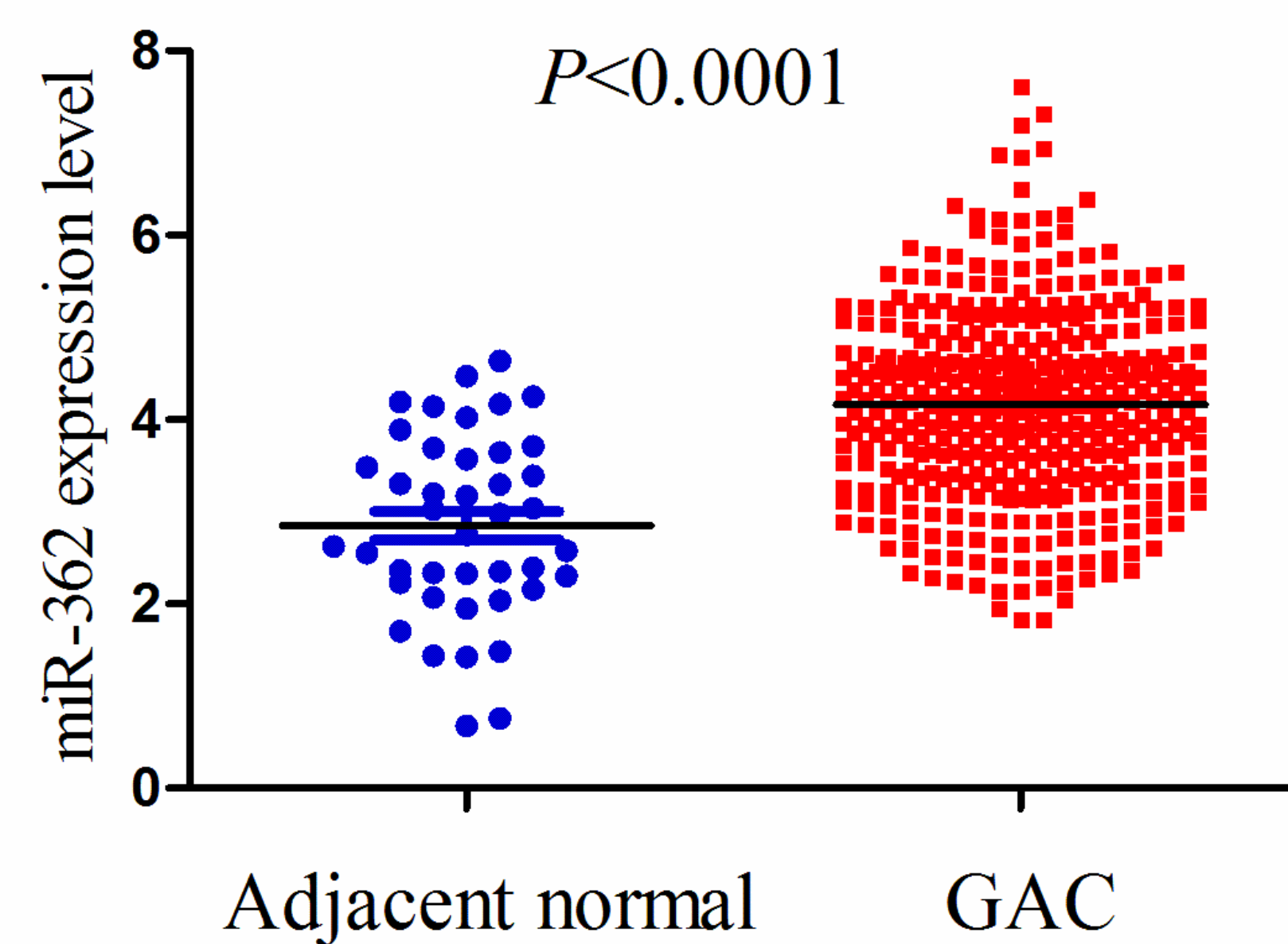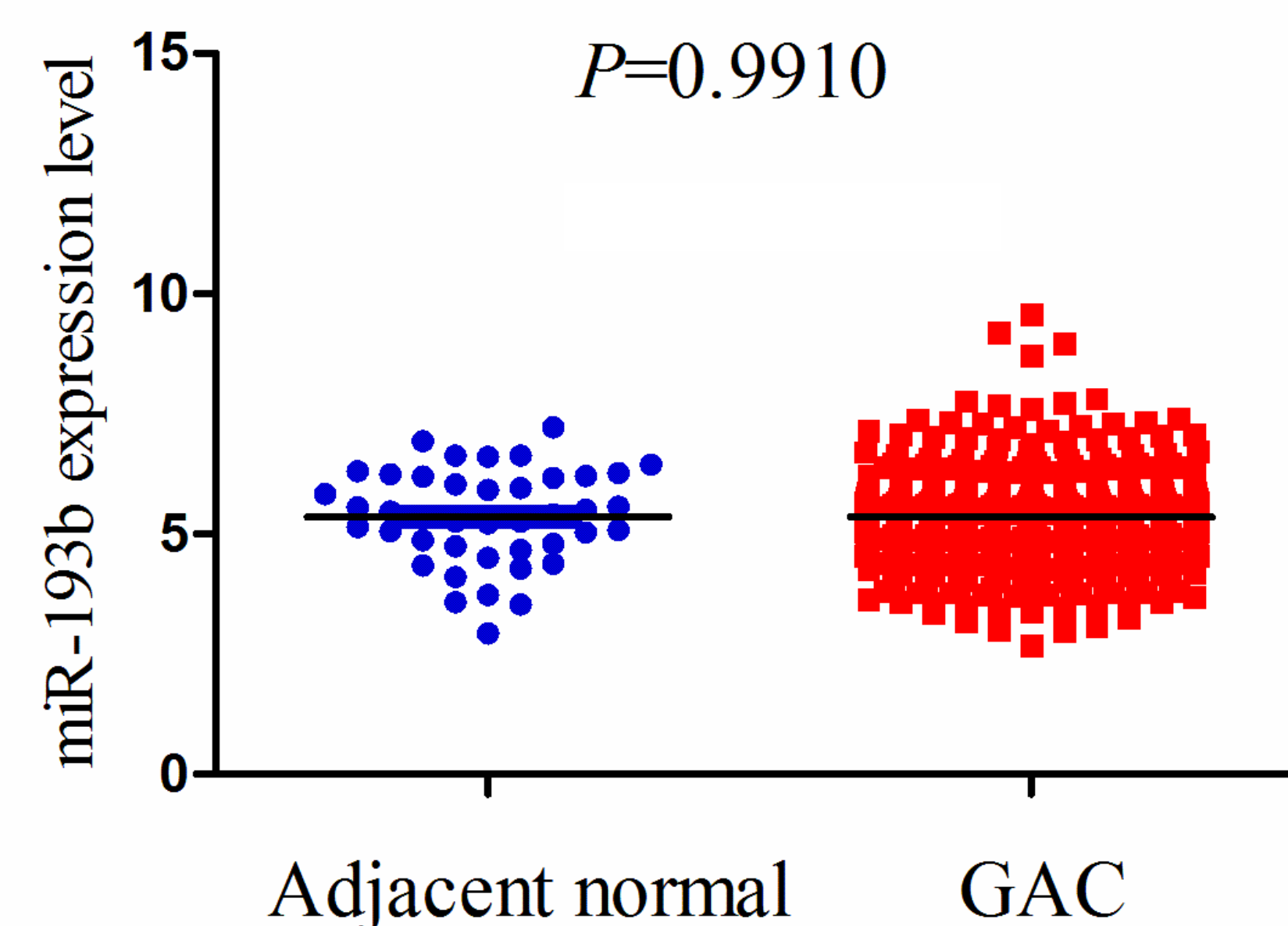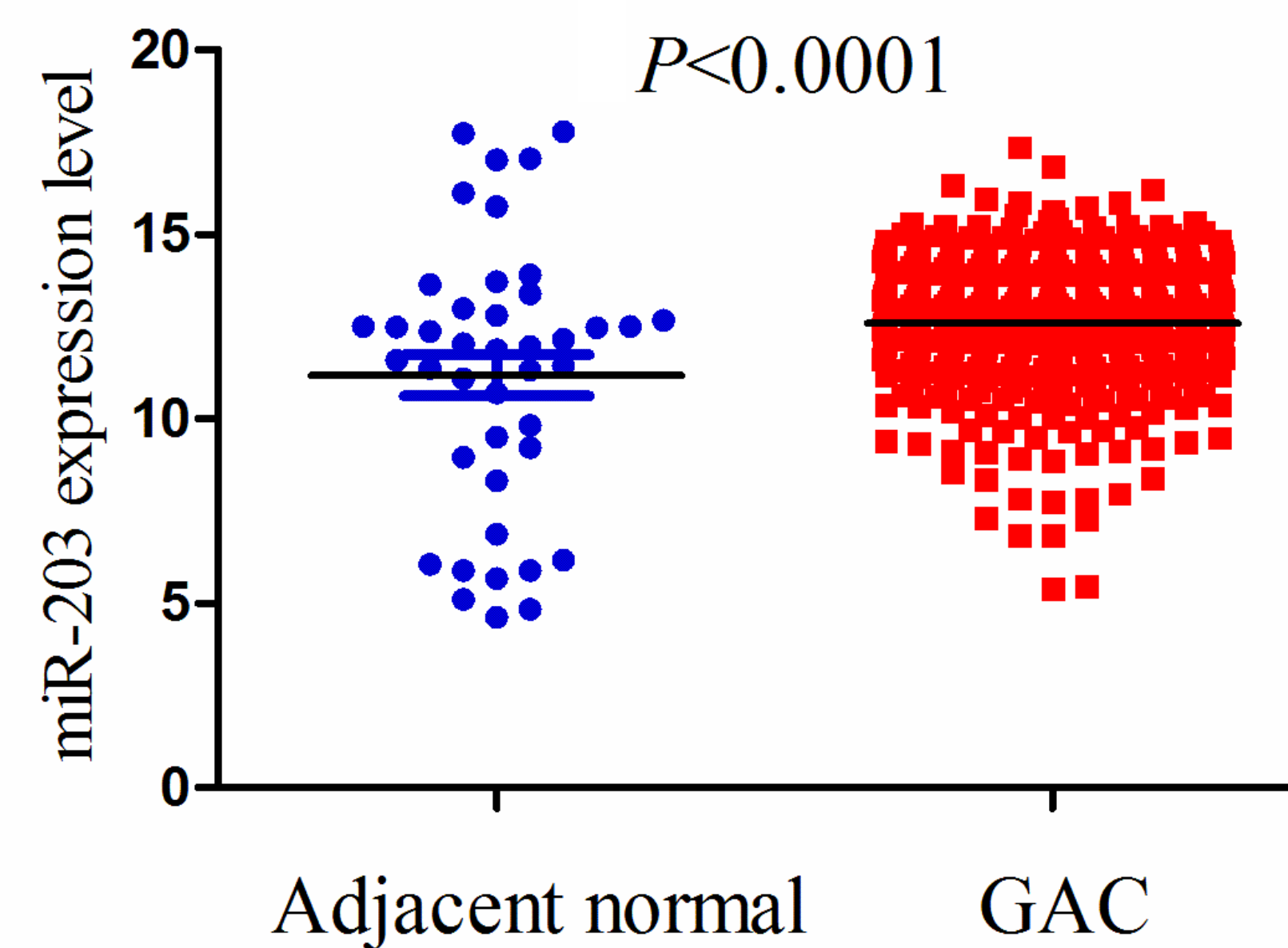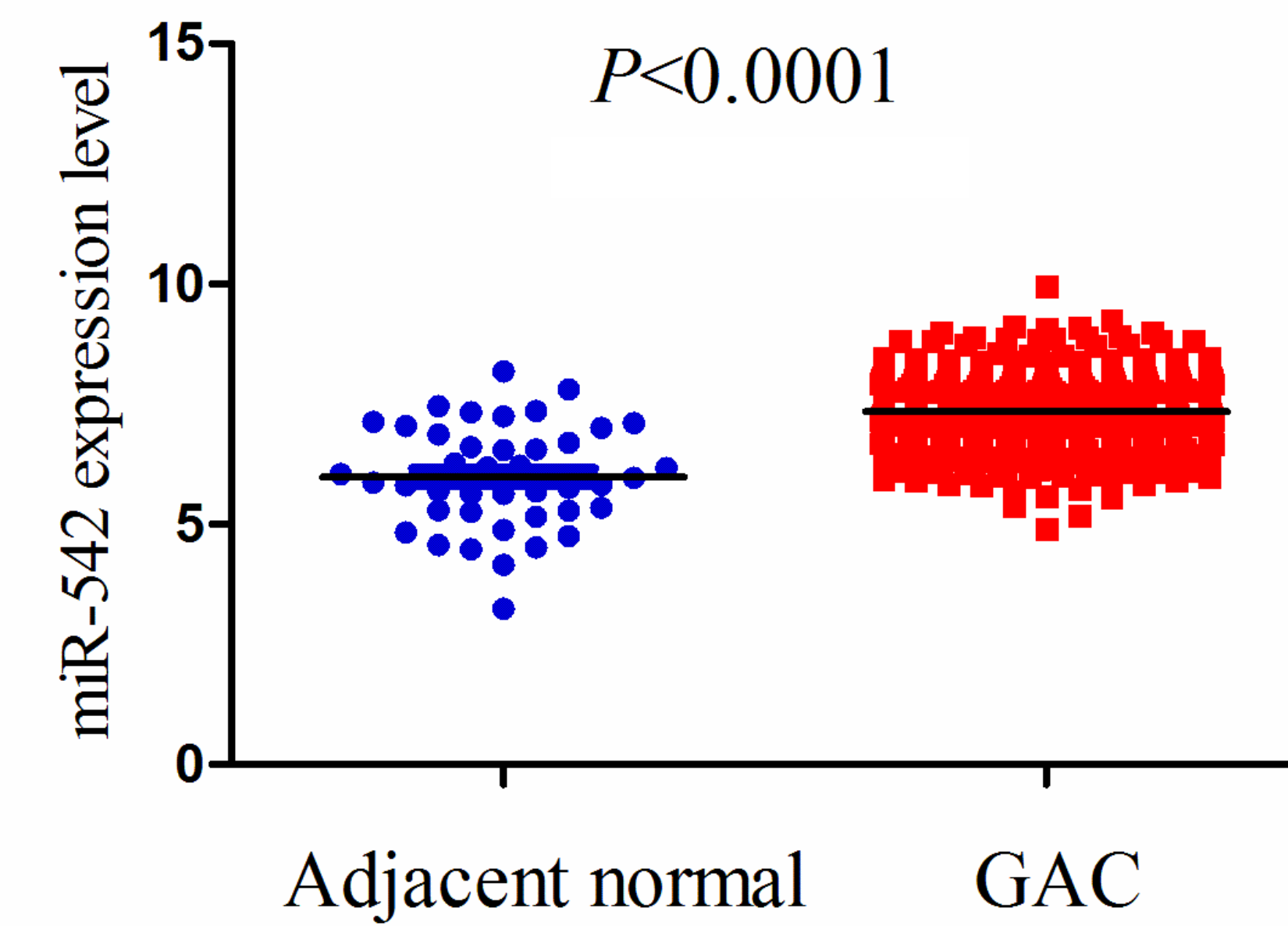

Supplement: Supplementary file 6 — Figure S5. TCGA analysis of the expression levels of miR-193b-5p, miR-542-3p, miR-362-5p and miR-203a-5p in paired and unpaired GC tissues. (PDF 229 kb) [file 12943_2019_1015_MOESM6_ESM.pdf]

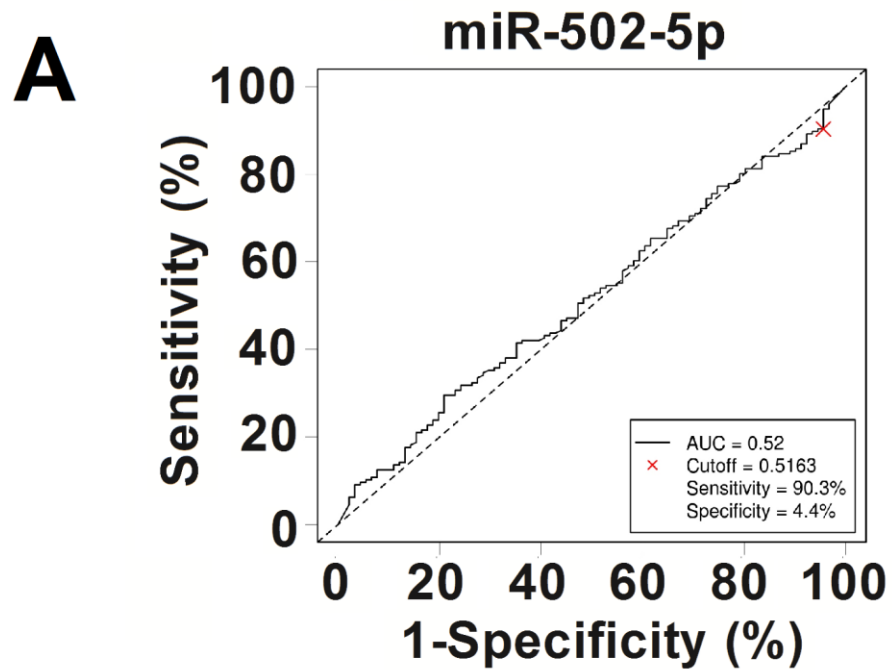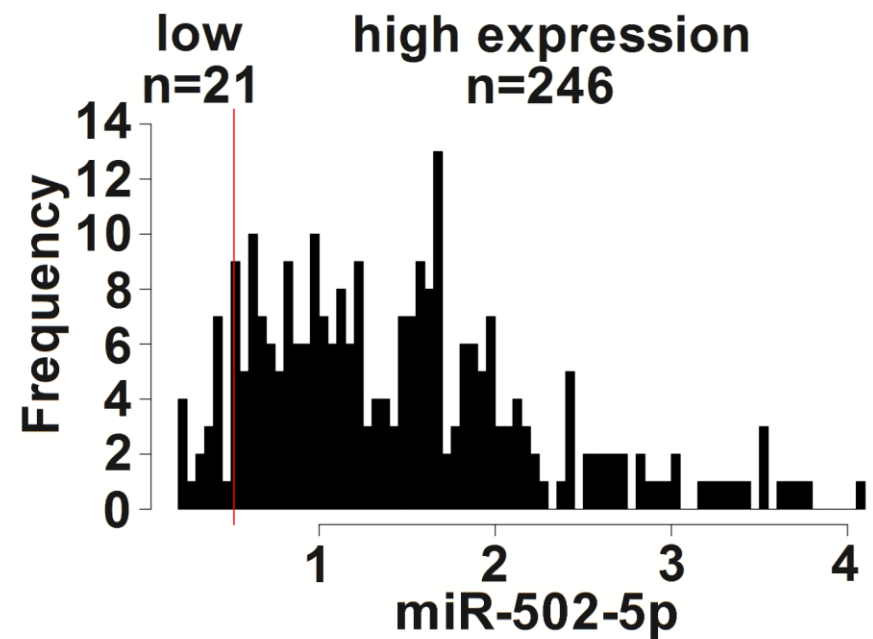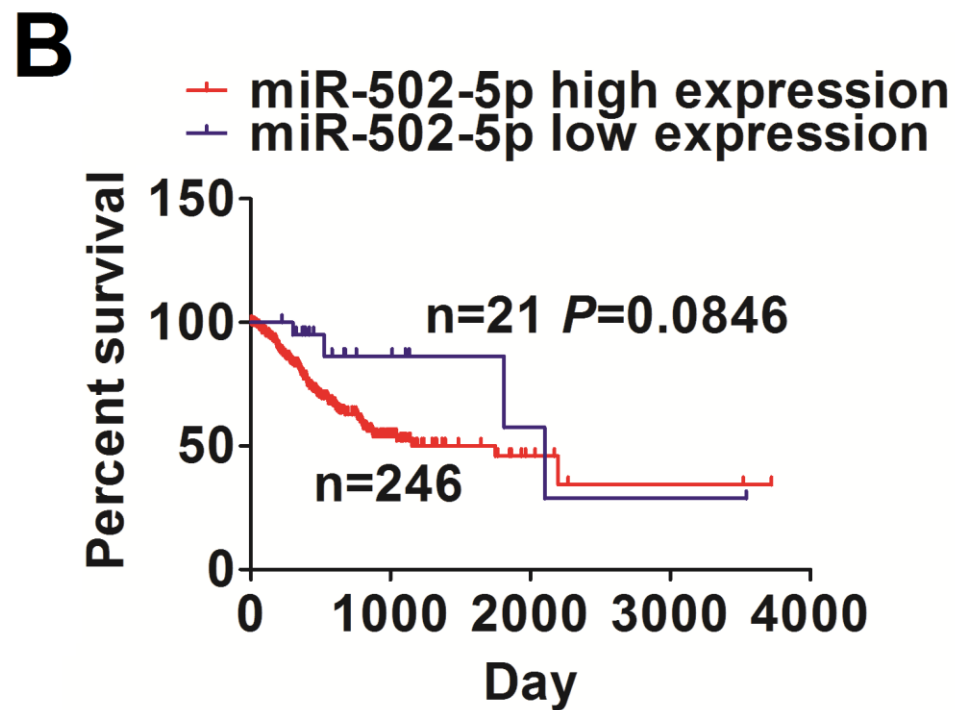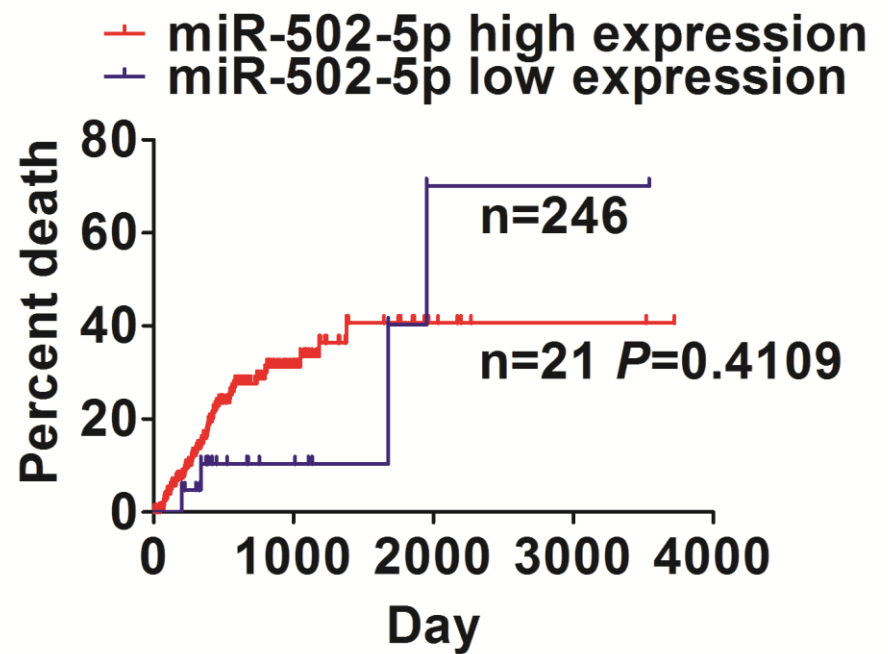

Supplement: Supplementary file 7 — Figure S6. TCGA analysis of the association of high or low miR-502-5p expression with the overall survival and tumor recurrence of GC patients. (PDF 343 kb) [file 12943_2019_1015_MOESM7_ESM.pdf]

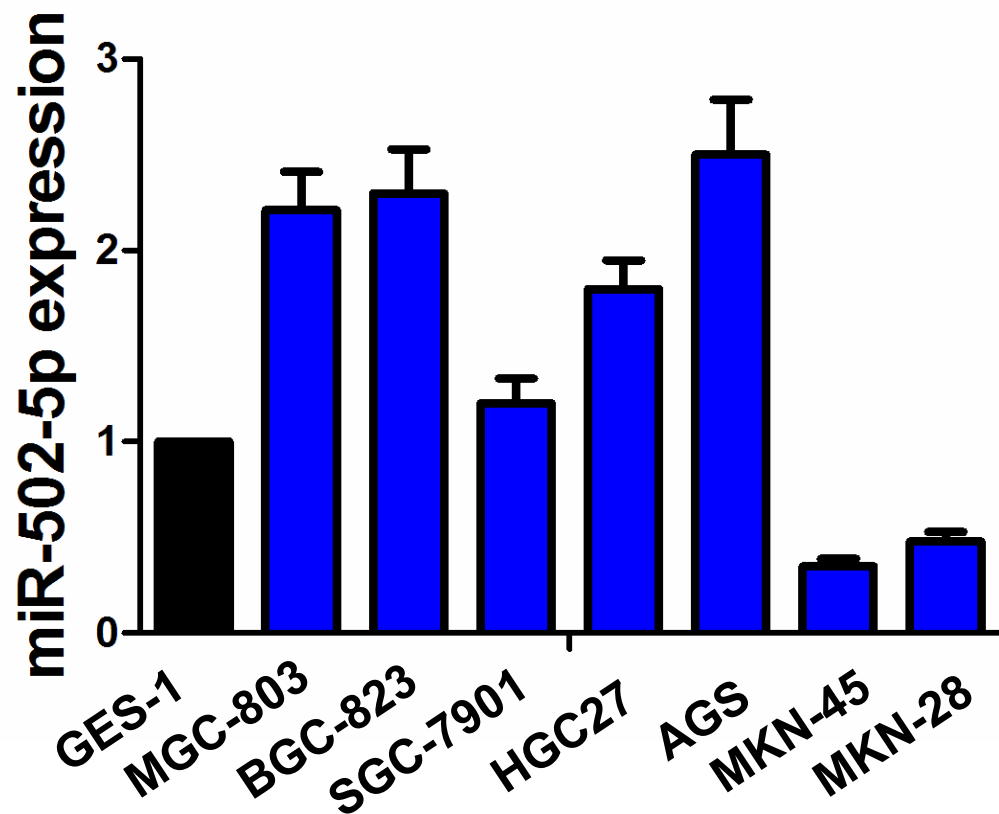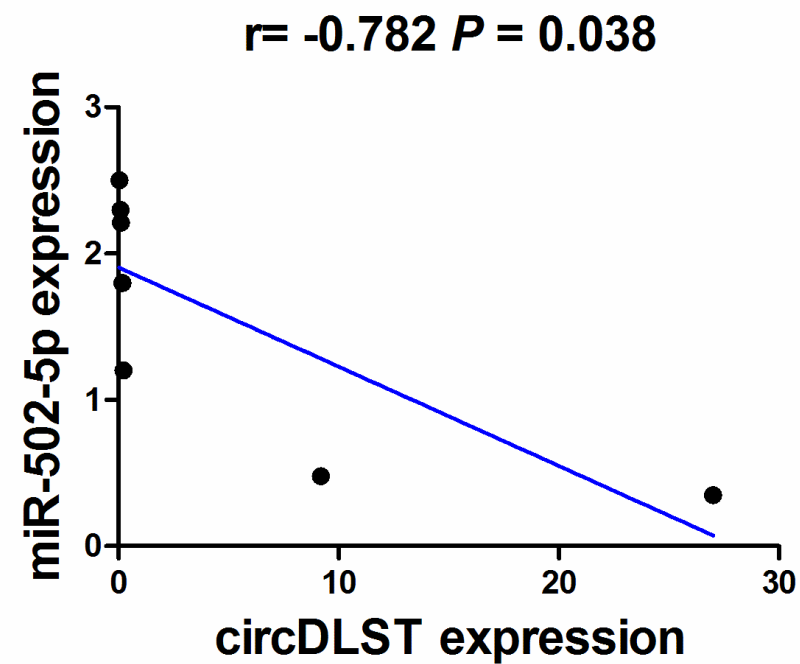

Supplement: Supplementary file 8 — Figure S7. qRT-PCR analysis of the expression levels of miR-502-5p and its correlation with circDLST in GC cell lines. (PDF 49 kb) [file 12943_2019_1015_MOESM8_ESM.pdf]

# FOXO SIGNALING PATHWAY

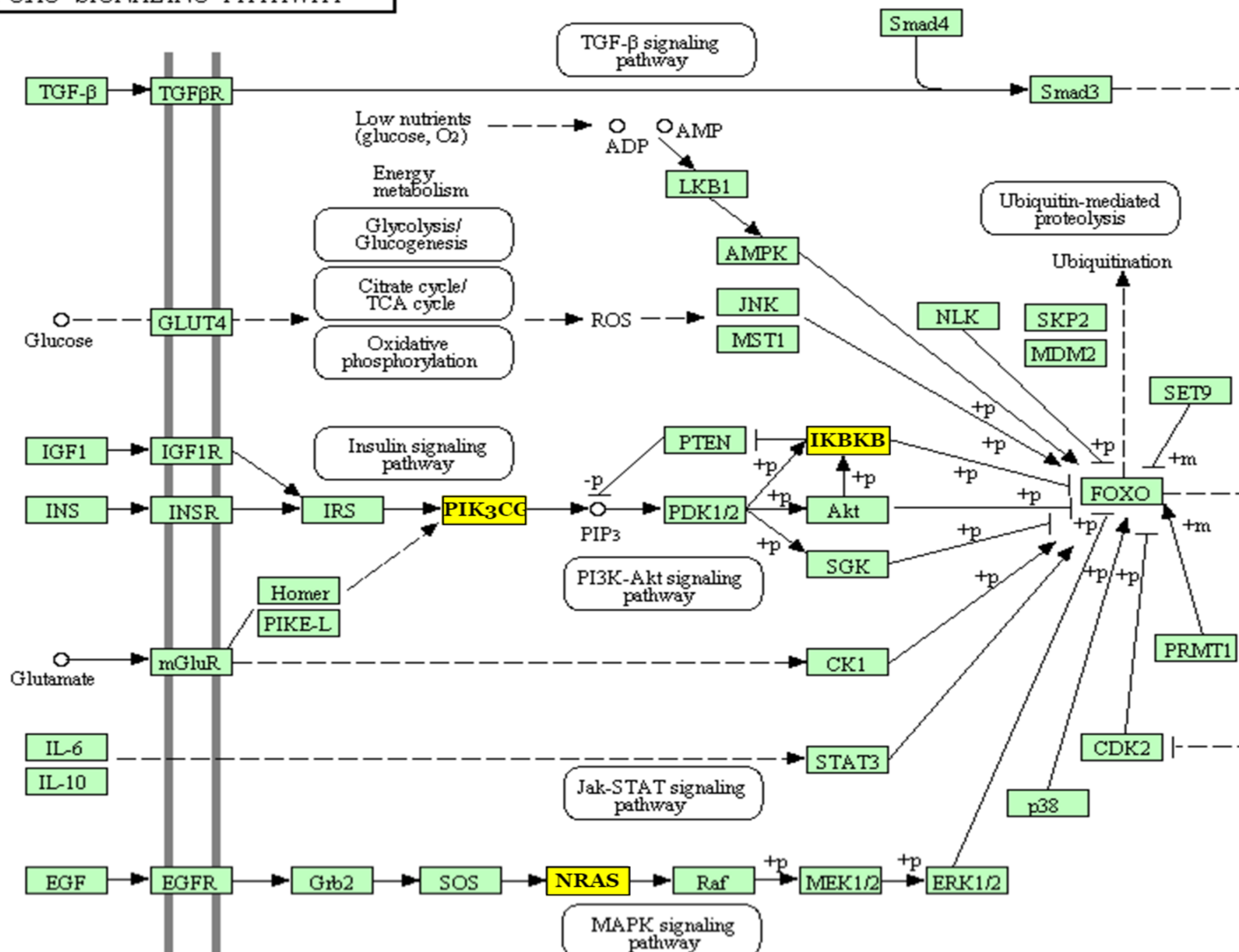

Supplement: Supplementary file 9 — Figure S8. Schematic representation of the involvement of NRAS in MEK/ERK signaling pathway. (PDF 1238 kb) [file 12943_2019_1015_MOESM9_ESM.pdf]

**A**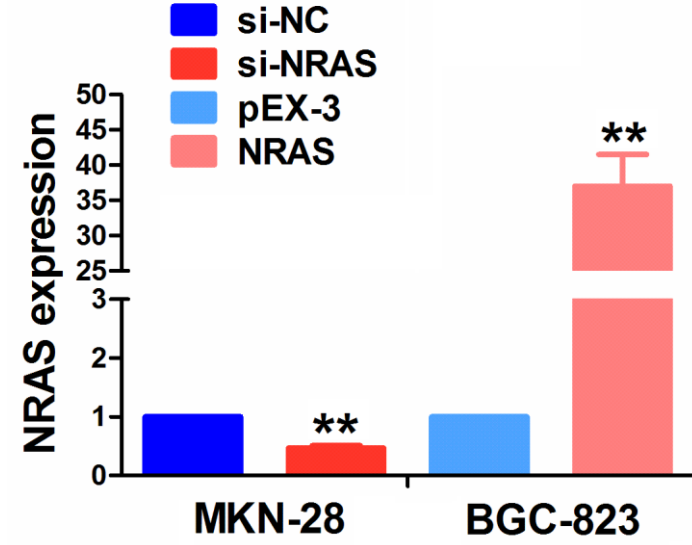**B**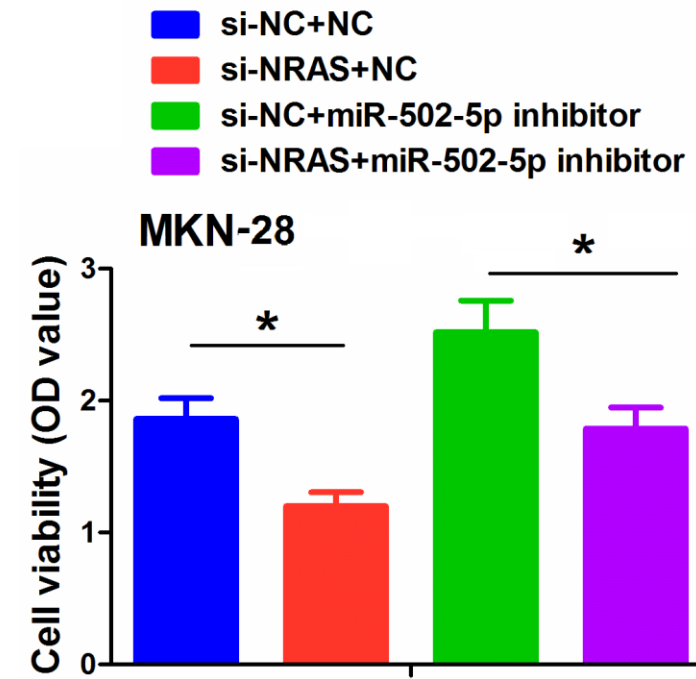**C**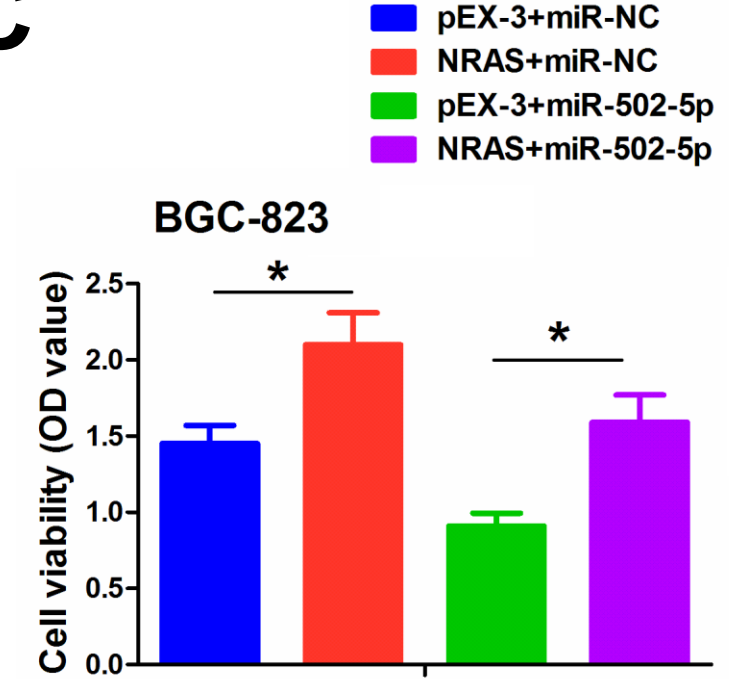**D**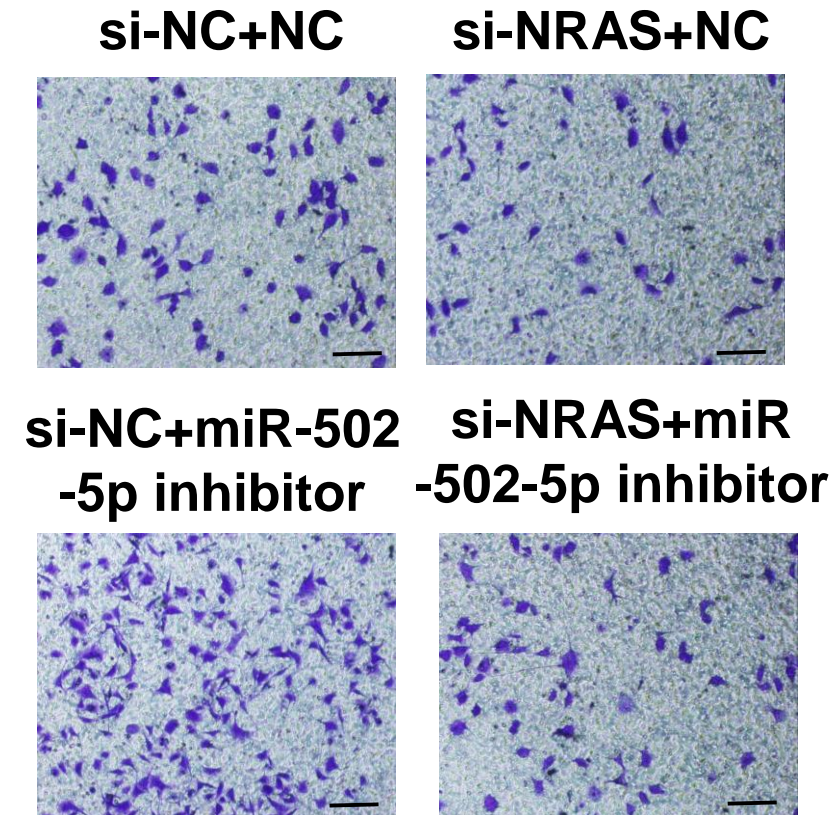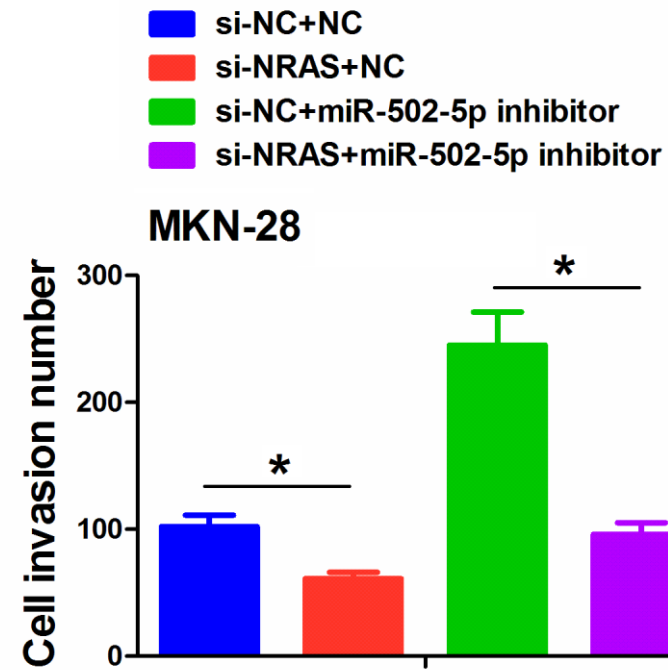**E**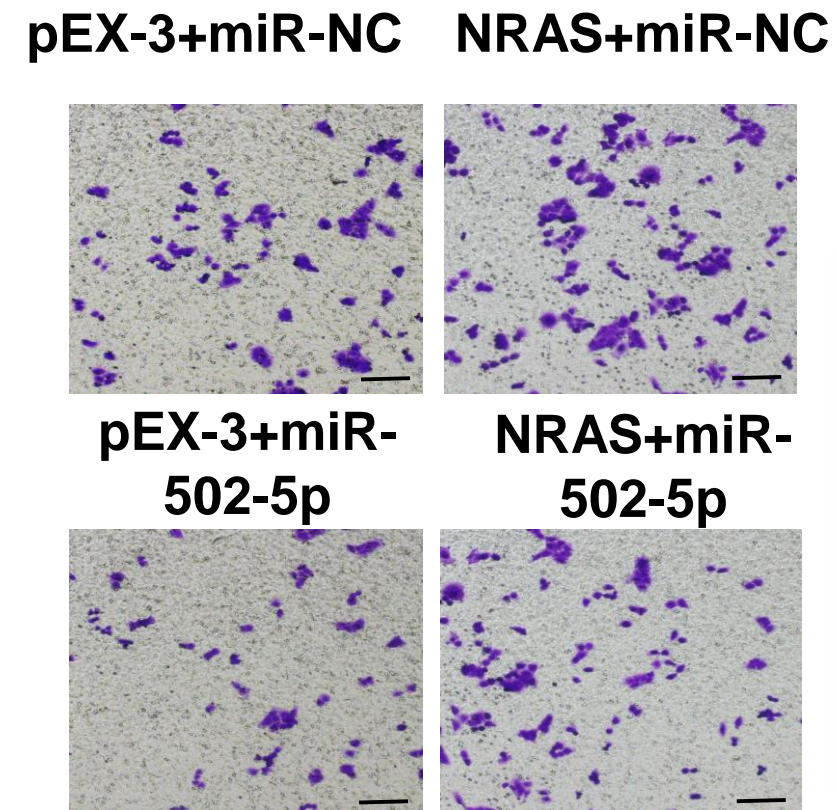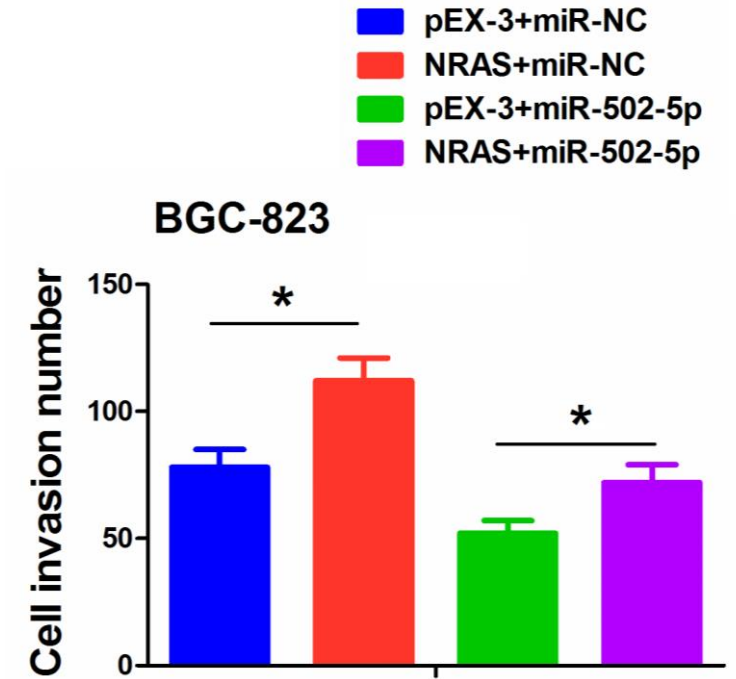

Supplement: Supplementary file 10 — Figure S9. NRAS reversed the tumor-suppressive effects of miR-502-5p in GC cells. (A) qRT-PCR and Western blot analysis of the transfection efficiency of si-NRAS or NRAS plasmid in MKN-28 or BGC-823 cells. (B-E) MTT and Transwell analysis of the cell viability and invasive potential after the co-transfection of miR-502-5p inhibitor and si-NRAS in MKN-28 cells or miR-502-5p mimic and NRAS in BGC-823 cells. Bar scale: 125 μm. Data are the means ± SEM of three experiments. *P < 0.05; **P < 0.01. (PDF 565 kb) [file 12943_2019_1015_MOESM10_ESM.pdf]
