# Supplementary material for: CircDLST promotes the tumorigenesis and metastasis of gastric cancer by sponging miR-502-5p and activating the NRAS/MEK1/ERK1/2 signaling
Source: Mol Cancer. 2019 Apr 5;18:80. doi: 10.1186/s12943-019-1015-1 (PMC6449953; doi:10.1186/s12943-019-1015-1)
Supplement: Supplementary file 5 — Figure S4. Schematic representation of potential binding sites of miRNAs with WT or MUT circDLST. (PDF 228 kb) [file 12943_2019_1015_MOESM5_ESM.pdf]

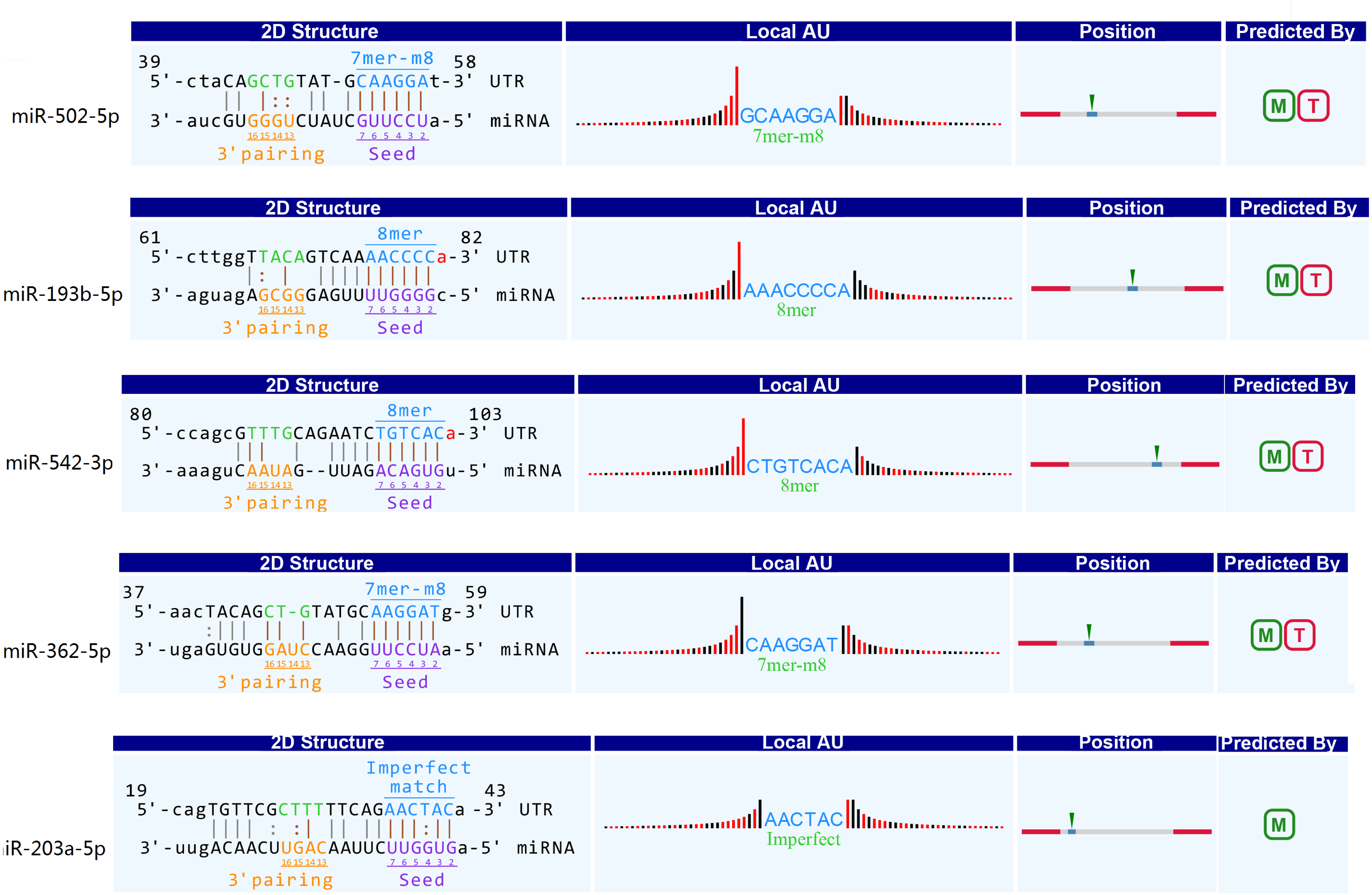
2D Structure
Local AU
Position
Predicted By
miR-502-5p
39
7mer-m8
58
5'-ctaCAGCTGTAT-GCAAGGAt-3' UTR
3'-aucGUGGGUCUAUCGUUCCUa-5' miRNA
16 15 14 13
7 6 5 4 3 2
3'pairing
Seed
GCAAGGA
7mer-m8
M
T
miR-193b-5p
61
8mer
82
5'-cttggTTACAGTCAAAACCCCa-3' UTR
3'-aguagAGCGGGAGUUUUGGGGc-5' miRNA
AAACCCCA
8mer
miR-542-3p
80
8mer
103
5'-ccagcGTTTGCAGAATCTGTCACa-3' UTR
3'-aaaguCAAUAG--UUAGACAGUGu-5' miRNA
CTGTCACA
8mer
miR-362-5p
37
7mer-m8
59
5'-aacTACAGCT-GTATGCAAGGATg-3' UTR
3'-ugaGUGUGGAUCCAAGGUUCCUAa-5' miRNA
CAAGGAT
7mer-m8
iR-203a-5p
19
Imperfect match
43
5'-cagTGTTCGCTTTTTCAGAACTACa -3' UTR
3'-uugACAACUUGACAAUUCUUGGUGa-5' miRNA
AACTAC
Imperfect
